# Motivated reasoning about climate change and the influence of Numeracy, Need for Cognition, and the Dark Factor of Personality

**DOI:** 10.1038/s41598-024-55930-9

**Published:** 2024-03-07

**Authors:** Fabian Hutmacher, Regina Reichardt, Markus Appel

**Affiliations:** 1 Human-Computer-Media Institute, Julius-Maximilians-University Würzburg, Oswald-Külpe-Weg 82, 97074 Würzburg, Germany; 2 Department of Psychology, University of Regensburg, Regensburg, Germany

**Keywords:** Psychology, Human behaviour

## Abstract

Human information processing is not always rational but influenced by prior attitudes, a phenomenon commonly known as motivated reasoning. We conducted two studies (*N*_*1*_ = 556, *N*_*2*_ = 1198; UK samples) investigating motivated reasoning in the context of climate change with a focus on individual differences as potential moderating factors. While previous research investigated motivated reasoning regarding the debate whether climate change is anthropogenic, we focused on current discourses about the effectiveness of different countermeasures. To this end, participants evaluated fictitious scientific data on the effectiveness of regulations to reduce CO_2_ emissions. In both studies, participants exhibited motivated reasoning as indicated by the observation that prior attitudes about CO_2_ reduction policies predicted evaluation of the scientific data. The degree of motivated reasoning was not related to individual difference variables, namely the ability to understand and reason with numbers (Numeracy), the willingness to show this ability (Need for Cognition), and the tendency to maximize one’s individual utility (Dark Factor of Personality). However, numeracy was associated with a less biased interpretation of the presented information. Our research demonstrates that motivated reasoning is a general phenomenon, and points to numerical training as one way to improve reasoning.

## Introduction

While there is wide scientific consensus on the anthropogenic nature of climate change^1^ and the need to act^2^, there is a vivid debate about the best and most effective ways of addressing the current global crisis. Crucially, this debate is not only relevant to scientists, activists, and politicians, but also to the public. In order to adjust their behavior and to demand political action, individuals need to understand the existing scientific evidence and the multilayered consequences of different mitigation policies^3,4^. However, the positions that individuals hold regarding these issues are overshadowed by their values and worldviews^5^, potentially undermining the rational discourse. As it seems, not everyone is willing to follow “the unforced force of the better argument”^6^, p. 305. In psychology, this is commonly known as *motivated reasoning*. Against this background, the present studies were aimed at investigating motivated reasoning in the context of climate change and the influence that different dimensions of individual differences have on the degree of biased reasoning. In particular, we tested whether motivated reasoning is influenced by the ability to arrive at a correct interpretation of the presented numerical information (Numeracy), the willingness to show this ability (Need for Cognition), and the tendency to maximize one’s individual utility (Dark Factor of Personality).

## Motivated reasoning

Motivated reasoning denotes the phenomenon that human information processing is shaped by the individual’s motives, goals, and attitudes instead of being rational and objective^7,8^. That is, “people are more likely to arrive at those conclusions that they want to arrive at”^8^, p. 495. When motivated reasoning manifests itself in the context of evaluating scientific evidence on a given topic, it is referred to as *motivated science reception* or *motivated rejection of science*^9–11^. Over the last decades, researchers have investigated various aspects of motivated reasoning. For instance, it has been shown that individuals tend to seek out information that confirms their prior attitudes^12,13^. In addition, attitudes drive how individuals deal with new information that they are being confronted with: While information that is congruent with one’s attitudes is readily being accepted (*motivated acceptance*), information contradicting one’s attitudes is evaluated more critically^14,15^ and may trigger the active search for further congruent information (*motivated rejection*)^16^. In many situations, it is the combination of motivated acceptance and motivated rejection that can be considered to lie at the heart of motivated reasoning. Motivated reasoning has been demonstrated on a wide range of topics, such as capital punishment^17^, pacifism^18^, and nanotechnology^19^.

Importantly, there are several studies investigating motivated reasoning in the context of climate change^20–29^. All of these studies have found evidence for motivated reasoning and have thus contributed to our understanding of the current debates on climate change and environmental policies. Nevertheless, there are certain limitations to be noted. To begin with, all above-mentioned studies except from one^26^ were based on US samples, limiting the cross-cultural validity of the results. Importantly, the strong partisan divide on climate change beliefs, which can be observed in the US^30^, does not equally translate to other countries around the globe. Arguably, the lack of sample diversity is particularly problematic as it is linked to two further limitations. First, many of the above-mentioned studies use political orientation as a proxy for climate change attitudes instead of directly measuring these attitudes^21,23,26,27,29^, which seems reasonable in a situation in which there is a strong polarization along party lines in a particular country. However, political orientation is an extremely broad category, making it difficult to determine what kind of goals or motives actually drive motivated reasoning in these situations^13,30,31^. Second, several of the above-mentioned studies were restricted to investigating motivated reasoning regarding the question whether climate change is anthropogenic or not^20–22,28^. Again, this seems reasonable given the relatively high percentage of individuals in the US denying the anthropogenicity of climate change. Taken together, it remains unclear whether the current state of evidence on the mechanisms and moderators of motivated reasoning is limited to highly identity-relevant categories such as political orientation and to heated debates about the anthropogenicity of climate change. At the same time, the current debates in the public and political realm in many countries go beyond the general question about anthropogenicity and involve discussing the pros and cons of specific policies. Therefore, it is important to investigate whether and under what conditions motivated reasoning occurs with respect to these issues. Finally, much of the support for motivated reasoning in the context of climate change is derived from correlational evidence (e.g., between political orientation and policy support or risk perception^21,24,25,27^) and is consequently not based on performance on an actual task with objectively correct or wrong responses. The latter has the advantage that it allows disentangling motivated reasoning and unbiased reasoning^26^.

Against this background, we conducted two studies on motivated reasoning in the context of climate change that account for these limitations. Hence, we recruited participants in a non-US English-speaking country (United Kingdom) in order to ensure the cross-cultural validity of the existing evidence. In addition, and building on a similar study design used for investigating motivated reasoning in the context of the COVID-19 pandemic^32^, we asked participants about their attitude towards stricter regulations to reduce CO_2_ emissions and presented them with the task to evaluate two fictitious studies, one demonstrating that such regulations lead to a decrease of CO_2_ emissions and one demonstrating that stricter regulations lead to an increase of CO_2_ emissions. The fact that the outcome of the fictitious studies was manipulated within-subjects distinguishes our study from previous attempts to study motivated reasoning in the context of climate change using a performance-related measure^26,29^. Importantly, manipulating the study outcome within-subjects enabled us to calculate an individual bias score and to relate this individual bias score to other factors potentially influencing the degree of motivated reasoning (for details, see the method section). More specifically, we calculated a *directional bias score*, indicating both the direction and the degree of biased reasoning. A correlation between the directional bias score and attitude indicates that individuals engage in motivated reasoning. Based on the extant literature describing motivated reasoning in various contexts, we formulated the following hypothesis regarding the directional bias score:

**H1**: People show motivated reasoning in the context of climate change. More specifically, people evaluate data on the effectiveness of stricter regulations to reduce C0_2_ emissions in line with their prior attitude towards such regulations.

Furthermore, we also calculated an *absolute bias score*, indicating the degree of bias irrespective of the direction. The absolute bias score reflects the extent to which participants give the same response to both studies. A high absolute bias score indicates that participants do not recognize that the studies in fact show opposite patterns (i.e., that one study shows an *increase* while the other study shows a *decrease* of C0_2_ emissions). A low absolute bias score indicates that participants are able to differentiate between the two fictitious studies and understand that the two studies show different outcomes. The absolute bias score can be seen as an indicator of biased and incorrect reasoning independently from the participants’ attitude. To be clear, the absolute bias score does not reflect motivated reasoning (in the sense of attitude-consistent reasoning). Analyzing the absolute bias score is interesting because it should be related to participants’ ability to understand and to reason with numbers (for more details see below)^32^.

The possibility to calculate a directional bias score as well as an absolute bias score within a single paradigm constitutes a crucial advantage of our method, as it enables us to investigate biased reasoning in more detail. More specifically, we can investigate the degree of attitude-consistent reasoning (i.e., the correlation of attitude and the directional bias score) and potential factors moderating this relationship (see below). At the same time, we can also investigate the degree of biased reasoning independently of the direction (i.e., the absolute bias score) and potential moderating factors (see below). While the former sheds light on motivated reasoning, the latter allows a better understanding of the general extent of biased or incorrect reasoning.

## Potential factors influencing motivated reasoning

**Figure 1:**
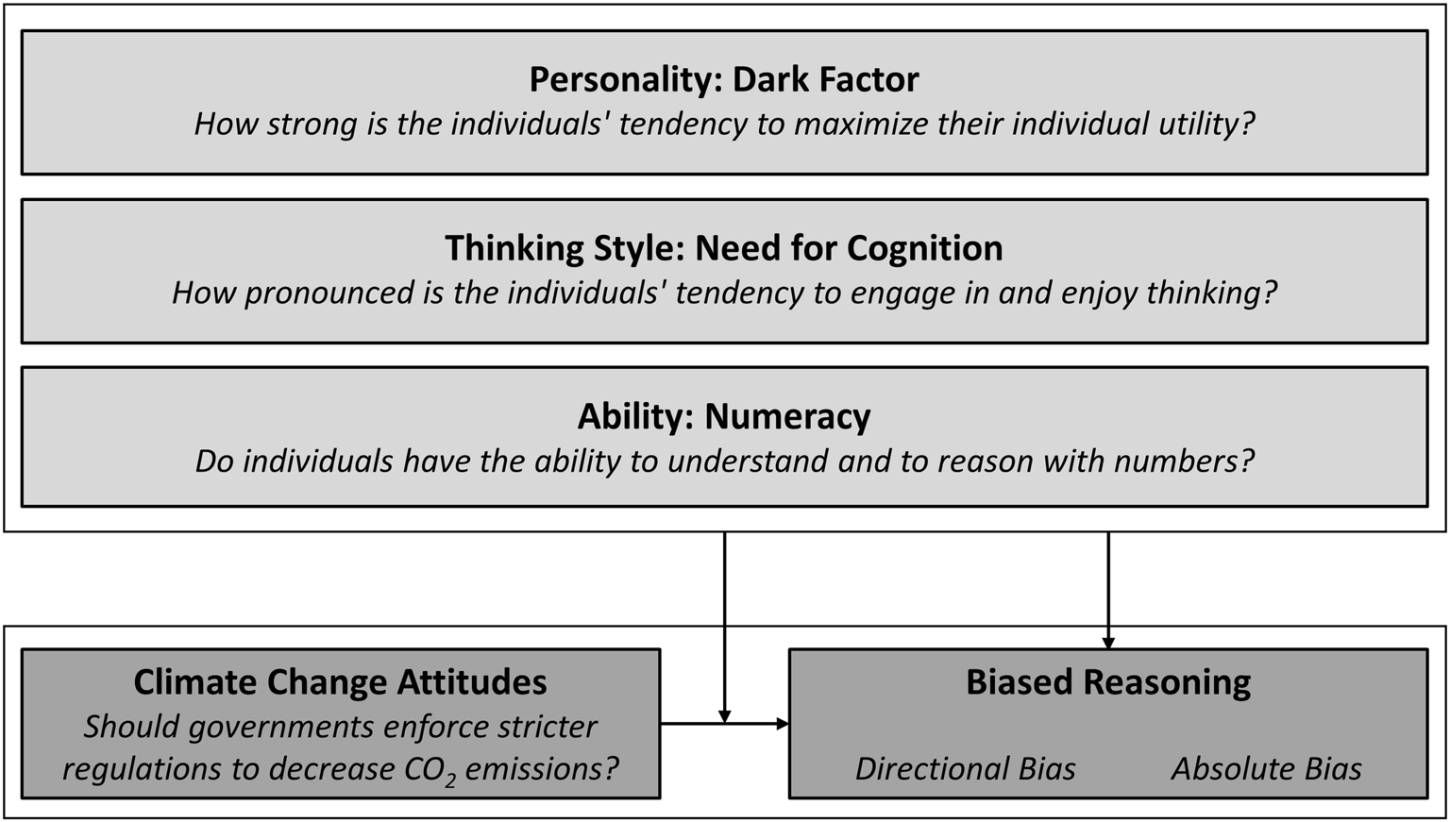
Study Overview. The present studies investigated the relationship between climate change attitudes and two forms of biased reasoning, as expressed in the directional bias and the absolute bias. In addition, it was tested whether this relationship as well as the degree of biased reasoning are influenced by three dimensions of individual differences: the ability to arrive at a correct interpretation of the presented information (*Numeracy*), the willingness to show this ability in a given situation (*Need for Cognition*), and the stable tendency to maximize one’s individual utility (*Dark Factor of Personality*).

Although motivated reasoning is generally considered to be a universal phenomenon, it is reasonable to assume that the degree of motivated reasoning depends on a wide range of boundary conditions^33^. In the context of the present studies, we sought to investigate three dimensions of individual differences that could potentially be related to the degree of motivated reasoning (for an overview, see Fig. 1). First, in order to arrive at an unbiased interpretation of new information, individuals need to have the necessary abilities to do so. As the fictitious studies that participants had to evaluate required them to reason with numbers, the ability component was operationalized in terms of *numeracy*. Second, even if individuals have the necessary abilities to arrive at an unbiased conclusion, they must be willing to use these abilities. Arguably, this willingness might depend on their thinking style as expressed through their *Need for Cognition*. Third, independently from their abilities and their willingness to use these abilities, the degree to which individuals engage in motivated reasoning might also depend on the general tendency to maximize one’s individual utility irrespective of the existing evidence, which is captured by the *Dark Factor of Personality*. These three dimensions of individual differences and the related research questions are described in detail below.

### Numeracy

*Numeracy* describes the ability to understand and to reason with numbers. Research has demonstrated that higher numeracy is associated with making better and more informed decisions^34,35^. For instance, individuals with higher numeracy have better financial literacy^36–38^ and better physical and mental health^39–41^. In the case of motivated reasoning, however, the picture is more complicated: On the one hand, it has been suggested that the degree of attitude-consistent judgment is more pronounced among highly numerate individuals, arguably because these individuals are better at finding rationalizations for their prior attitudes^21,25,26,42–44^. On the other hand, there are also studies which found no clear effects of numeracy on the degree of attitude-consistent judgment^45,46^ or even evidence in favor of reduced attitude-consistent judgment among highly numerate individuals^47,48^, providing support for the idea that numeracy can be an antidote to inaccurate and biased reasoning. The assumed reason behind such findings is that numeracy should enable individuals to consider the available evidence instead of simply falling for processes of motivated acceptance and motivated rejection. Note also that a study using the same procedure as the present investigation^32^ found no effect of numeracy on the degree of attitude-consistent judgment but observed that higher numeracy was associated with a reduced absolute bias. In other words, this study suggests that it may be worth investigating different kinds of biased reasoning in order to paint a nuanced picture of the role of numeracy. Given the two opposite theoretical perspectives and conflicting evidence, we formulated the following research question:

**Research Question 1**: Is the individual’s ability to understand and to reason with numbers (i.e., numeracy) related to (a) the degree of attitude-consistent judgment (i.e., the relation between attitude and directional bias) and (b) to the degree of absolute bias?

### Need for Cognition

*Need for Cognition* (NFC) is defined as “the tendency for an individual to engage in and enjoy thinking”^49^. Individuals with a high NFC prefer complex and intellectually challenging to simple problems, while individuals with a low NFC tend to avoid tasks that require deliberation and mental effort^50,51^. In line with this, it has been demonstrated that individuals with a high NFC show a higher interest in science^52^ and are more likely to be driven by accuracy goals compared to individuals with a low NFC^53^, suggesting that motivated reasoning might be reduced among individuals with a high NFC, arguably because these individuals are motivated to evaluate the available evidence thoroughly instead of simply accepting or rejecting it based on their prior attitudes. At the same time, evidence also suggests that individuals with a high NFC tend to have stronger and less ambivalent attitudes that are more resistant to change^54,55^, arguably precisely because of their tendency to base their opinions on an effortful analysis of the available evidence^51^, which supports the idea that a high NFC might be associated with more pronounced motivated reasoning under certain circumstances. In the context of climate change, studies suggest a link between a higher NFC and a higher probability to accept the anthropogenicity of climate change^56^ as well as more pronounced pro-environmental attitudes and goals^57^. However, a recent study that directly tested the influence of NFC on the degree of motivated reasoning in the context of climate change did not find any significant effects^20^. Given the ambiguous empirical evidence and given the theoretical plausibility of different outcomes, we formulated the following research question:

**Research Question 2**: Is the individual’s Need for Cognition related to (a) the degree of attitude-consistent judgment (i.e., the relation between attitude and directional bias) and (b) to the degree of absolute bias?

### Dark Factor of Personality

The *Dark Factor of Personality* (D) describes “the general tendency to maximize one’s individual utility – disregarding, accepting, or malevolently provoking disutility for others – accompanied by beliefs that serve as justifications”^58^. D is conceptualized to capture the common variance of dark personality traits such as Machiavellianism, egoism, and psychopathy^58,59^. It has been observed that individuals high in D hold problematic epistemic beliefs in the sense that they have a strong conviction that truth is political and ultimately a matter of power, that they believe that they can trust their intuition when evaluating facts, and that they have a low need to ensure that their beliefs align with the available evidence; in turn, these epistemic beliefs are associated with embracing conspiracy theories, a reduced ability to discern fake news from real news, and a lower probability of following public health recommendations in the context of the COVID-19 pandemic^60–62^. To our knowledge, D has not been investigated in the context of motivated reasoning and/or climate change. Given the above-mentioned evidence and given that scoring high on the dark triad (i.e., a related albeit distinct personality construct) is associated with a weaker conviction that climate change is anthropogenic^63^ as well as reduced pro-environmental behavioral intentions^64^, it nevertheless seems plausible to assume that D might play a role in the context of the present studies. In particular, one might assume that individuals high in D willingly accept information that suits their agenda and reject information that does not. As there is no direct evidence for this hypothesis yet, however, we formulated the following open research question:

**Research Question 3**: Is the individual’s Dark Factor of Personality score related to (a) the degree of attitude-consistent judgment (i.e., the relation between attitude and directional bias) and (b) to the degree of absolute bias?

## Study 1

### Method

#### Ethics statement

In Germany, where the principal investigators are located, ethics approval is deemed unnecessary according to national regulations for studies as the present ones^65^. Nevertheless, both Study 1 and Study 2 were conducted in full accordance with the Declaration of Helsinki, as well as the ethical guidelines provided by the German Psychological Society (DGPs)^66^. Of course, this also included obtaining informed consent from all participants before they were able to take part in this study.

#### Participants

As correlations between two variables can be assumed to stabilize at about 250 participants^67^ and as it has been recommended to at least double sample size when investigating interactions with an additional factor^68^, we aimed for a final sample size in Study 1 of at least 500 participants. Participants were recruited via Prolific (www.prolific.com). All data exclusions, manipulations, and measures are reported. The study was programmed on SoSciSurvey^69^.

##### Session 1: prescreening

In Session 1, participants were prescreened for their attitude towards stricter regulations to reduce CO_2_ emissions. As we were not sure how attitude would be distributed among participants and as we wanted to make sure to be able to invite a reasonable number of participants on each point of the attitude spectrum for the main study, we decided to collect data from at least 900 participants. In total, 902 participants completed the prescreening. Participation was only possible for Prolific users with an approval rate of at least 98% who were fluent in English and currently living in the United Kingdom. Participants were compensated with £1.70. Session 1 lasted about ten minutes. Data collection took place from August 11 to August 12, 2022.

##### Session 2: main study

To account for exclusions and potential dropout between sessions, we decided to invite 721 participants for Session 2. An equal distribution of participants across the seven scale points of the regulation attitude item would have implied inviting 103 participants per scale point for Session 2. Whenever we had 103 or fewer participants on one scale point of the regulation attitude item, all of these participants were invited for Session 2; whenever we had more than 103 participants on one of the seven points of the scale, we randomly drew a subset of participants (for more information about the participants who were selected for participation in Session 2 see the Supplemental Material S2 and S3).

From the total of 721 participants that were invited to participate in Session 2, 673 participants completed the study. Participants who did not pass all attention checks (*N* = 98) were excluded from the analysis. We also excluded participants who had noticeable short (i.e., less than one third of the median response time; *N* = 14) or long (i.e., more than six times the median response time; *N* = 5) response times, indicating careless responding. Thus, the final sample consisted of 556 participants (*M*_*age*_ = 43.40, *SD* = 14.05, 18–80 years; 226 male, 323 female, 3 other, 4 prefer not to say; see Supplemental Material S4–S6 for information about political orientation, ethnicity, and education). Participants were compensated with £1.05 upon study completion. Session 2 lasted about six minutes. Data collection took place from August 16 to August 18, 2022.

#### Materials

##### Attitude towards stricter regulations to reduce CO_2_ emissions

The attitude towards regulations to reduce CO_2_ emissions was measured using one item created for the purpose of the study (“Governments should enforce stricter regulations to decrease CO_2_ emissions, even if these regulations restrict the freedom of the individual”). We additionally assessed three facets of attitude strength (all items were measured on 7-point Likert-type scales ranging from 1 to 7)^70^: *attitude certainty* (“How certain are you of your views about regulations to reduce CO_2_ emissions?” “How sure are you that your opinion about regulations to reduce CO_2_ emissions is right?”), *attitude importance* (“To you personally, how important is the issue of regulations to reduce CO_2_ emissions?” “Personally, how much do you care about the issue of regulations to reduce CO_2_ emissions?”), and *ego-involvement* (“How central is your attitude toward regulations to reduce CO_2_ emissions to your self-image?” “How representative of your values is your attitude toward regulations to reduce CO_2_ emissions?”). The internal consistency of the 6-item attitude strength scale was good, α = 0.85. As they were not part of the main research questions and yielded no significant results, the analyses regarding attitude strength are reported in the Supplemental Material S8 (Study 1) and S19 (Study 2).

##### Studies on the effectiveness of stricter regulations to reduce CO_2_ emissions

**Figure 2:**
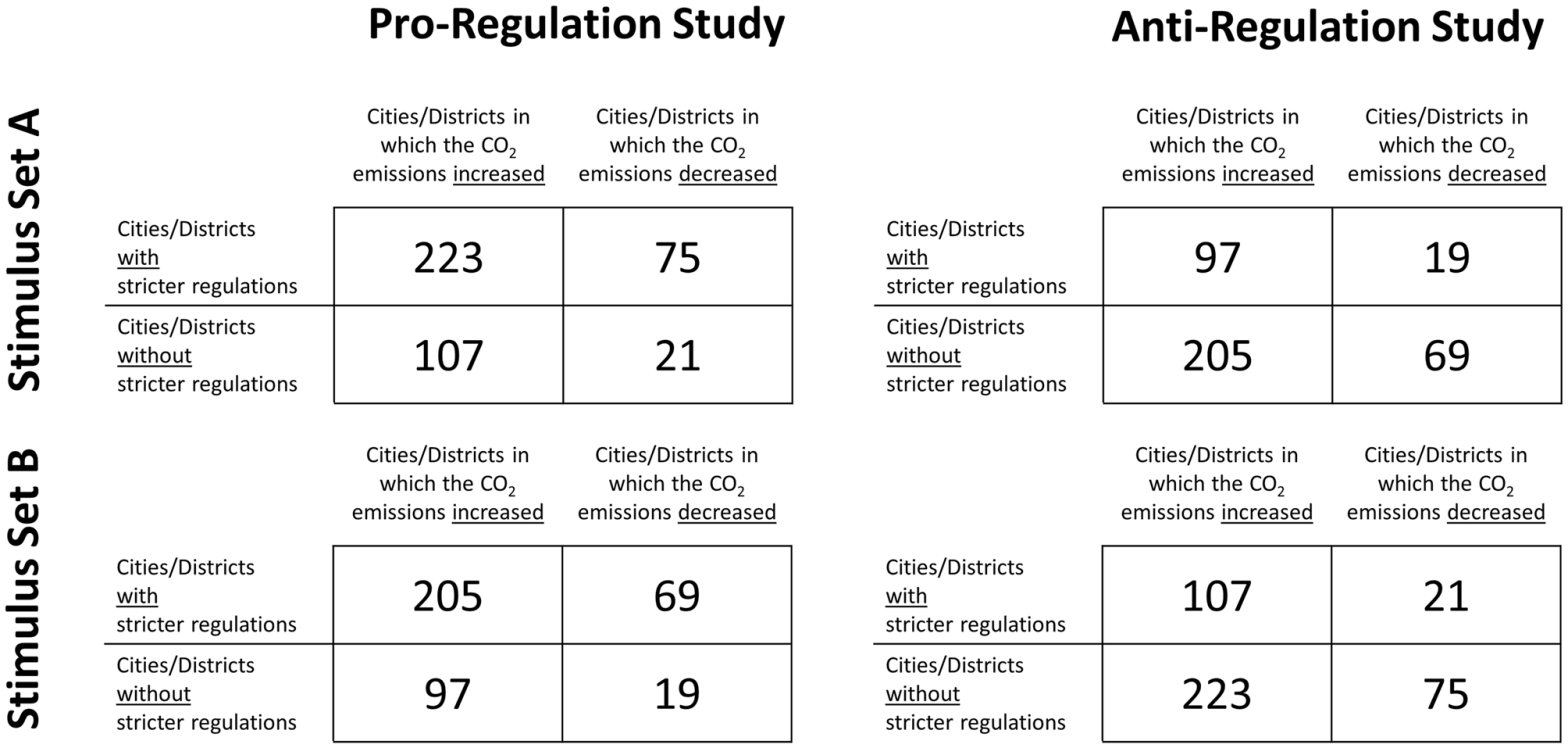
Study Conditions. Each participant evaluated the results of two fictitious studies: one study demonstrating that stricter regulations to reduce CO_2_ emissions are effective (*pro-regulation study*) and one study demonstrating that stricter regulations to reduce CO_2_ emissions are counterproductive (*anti-regulation study*). Which of the two sets of numbers was used for the pro-regulation study and for the anti-regulation study was counterbalanced across participants (Stimulus Set A vs. Stimulus Set B). Whether participants first evaluated the pro-regulation study or the anti-regulation study was counterbalanced across participants.

Participants evaluated two fictitious studies on the effectiveness of stricter regulations to reduce CO_2_ emissions (see Fig. 2): one study demonstrating that stricter regulations to reduce CO_2_ emissions are effective (*pro-regulation study*) and one study demonstrating that stricter regulations to reduce CO_2_ emissions are counterproductive (*anti-regulation study*). The results of the studies were presented in two-by-two contingency tables adapted from a previous study^32^. As the study outcome was manipulated within-subjects, two different sets of numbers were used. These are designed in a way so that the Binomial Effect Size Display (BESD)^71^ is equivalent across conditions (BESD = 8.8% in the pro-regulation studies, BESD = -8.8% in the anti-regulation studies)^72^. Furthermore, all contingency tables show that there were more cities/districts in which the CO_2_ emissions increased (77%) than cities/districts in which the CO_2_ emissions decreased (23%). Thus, the overall trend was held constant across the two sets of numbers.

Most importantly, the numbers in the contingency tables were designed in a way so that superficial processing (i.e., comparing absolute numbers between two cells instead of ratios) easily leads to the wrong interpretation^43^. Take the pro-regulation study in Stimulus Set A, for instance: When looking at the absolute numbers in the upper left and lower left cell only, there are more cities/districts with stricter regulations to reduce CO_2_ emissions (223) than cities/districts without stricter regulations to reduce CO_2_ emissions (107) that show an increase in CO_2_ emissions, which can lead to the wrong conclusion that stricter regulations are counterproductive. In fact, however, there is an increase in CO_2_ emissions in 223 out of 298 schools with stricter regulations (74.8%) compared to an increase in CO_2_ emissions in 107 out of 128 cities/districts without stricter regulations (83.6%), indicating that stricter regulations are an overall successful intervention. The logic behind the numbers in the anti-regulation study is exactly the same, leading to the opposite conclusion (for details, see the Supplemental Material S11).

**Figure 3:**
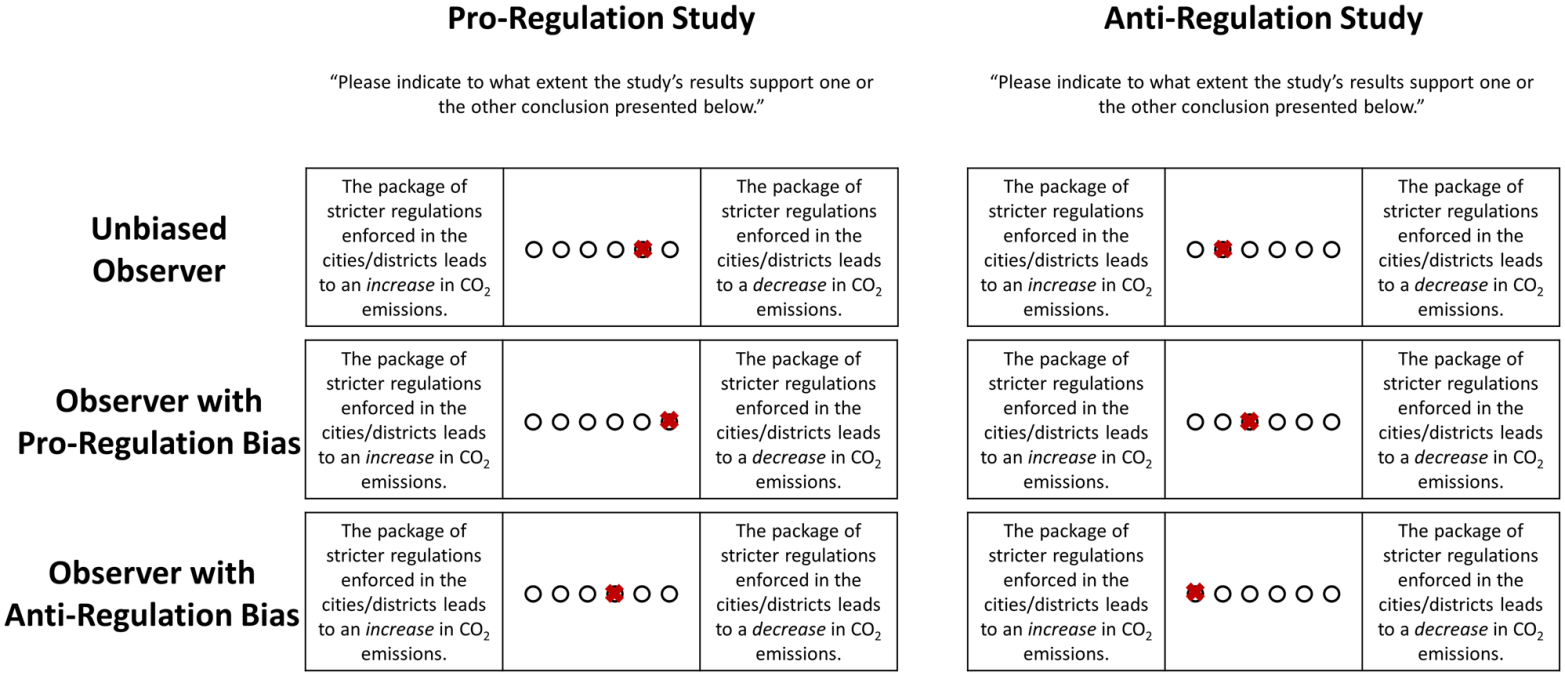
Bias Scores. The figure depicts three potential response patterns of an unbiased observer, an observer with a pro-regulation bias, and an observer with an anti-regulation bias. The *anti-regulation/pro-regulation bias score* or *directional bias score* is the difference between the response to the pro-regulation study and the reversely coded response to the anti-regulation study (unbiased observer: 5–5 = 0; observer with pro-regulation bias: 6–4 = 2; observer with anti-regulation bias: 4–6 = –2). The directional bias score indicates both the direction and the degree of the bias. By taking the absolute value of the directional bias score, one can calculate an *absolute bias score*, which indicates the degree of the bias irrespective of the direction (unbiased observer: 0, observer with pro-mask bias: 2, observer with anti-mask bias: 2).

Participants were asked to indicate what can be concluded from the presented studies on a 6-point Likert scale (ranging from 1 = “The package of stricter regulations enforced in the cities/districts leads to an *increase* in CO_2_ emissions” to 6 = “The package of stricter regulations enforced in the cities/districts leads to a *decrease* in CO_2_ emissions”). Note that participants may legitimately differ regarding their estimation of the strength of the evidence provided by the studies (i.e., response options 4–6 are correct in the case of the pro-regulation study and response options 1–3 are correct in the case of the anti-regulation study). As the ratios in the pro-regulation and the anti-regulation study are exactly reversed and the BESD is equivalent (see above), however, an unbiased observer should show a symmetric response pattern (e.g., tick response option “4” when evaluating the pro-regulation study and option “3” when evaluating the anti-regulation study). Differently put, an asymmetric response pattern indicates a bias (see Fig. 3).

Based on this assumption, two dependent variables can be calculated. The *anti-regulation/pro-regulation bias score* or *directional bias score* is the difference between the response to the pro-regulation study and the reversely coded response to the anti-regulation study. A positive value indicates a pro-regulation bias, while a negative value indicates an anti-regulation bias. More extreme values indicate a more extreme bias. That is, the directional bias score indicates both the direction and the degree of the bias. As the directional bias score combines the participants’ response to attitude-consistent and attitude-inconsistent information, it mirrors the joint effects of motivated acceptance and motivated rejection. In case participants engage in motivated reasoning in the context of climate change (i.e., show attitude-consistent judgment), individuals who support stricter regulations to reduce CO_2_ emissions should on average show a positive directional bias while individuals who are against stricter regulations should on average show a negative directional bias. Note that a zero directional bias score does not necessarily indicate a correct evaluation of the studies, as this score can also result from a symmetric, but incorrect evaluation of the studies. By taking the absolute value of the directional bias score, one can calculate an *absolute bias score*, which indicates the degree of the bias irrespective of the direction. That is, the absolute bias score mirrors the degree of asymmetry when evaluating the two fictitious studies. In other words, it mirrors whether participants are able to differentiate between the two fictitious studies and whether they understand that the two studies show different outcomes. In case participants supporting and participants opposing stricter regulations to reduce CO_2_ emissions are equally biased, however, there will be no association between attitude and absolute bias. Apart from that, one can calculate the study evaluation accuracy, i.e., whether a study is evaluated correctly (1) or not (0).

##### Numeracy

Numeracy was measured using an 8-item scale^73^. The scale includes items such as “In the Big Bucks Lottery, the chances of winning a $10.00 prize are 1%. What is your best guess about how many people would win a $10.00 prize if 1,000 people each buy a single ticket from Big Bucks?”. The numeracy score for each participant equals the number of correct responses, ranging from zero to eight.

##### Need for Cognition

NFC was measured using an 18-item scale^74^. The scale includes items such as “I would prefer complex to simple problems” or “I find satisfaction in deliberating hard and for long hours”. Items were rated on a 9-point Likert scale ranging from 1 (very strong disagreement) to 9 (very strong agreement). The internal consistency of the 18-item scale was excellent, α = 0.94.

##### Dark Factor of Personality

D was measured using an 16-item scale^75^. The scale includes items such as “My own pleasure is all that matters” or “Payback needs to be quick and nasty”. Items were rated on a 5-point Likert scale ranging from 1 (strongly disagree) to 5 (strongly agree). The internal consistency of the 16-item scale was good, α = 0.85.

#### Procedure

##### Session 1: prescreening

After providing informed consent, participants answered demographic questions regarding age, gender, political orientation, ethnicity, and education. Next, participants were asked about their attitude towards stricter regulations to reduce CO_2_ emissions, followed by the scales measuring the NFC, D, and numeracy. Session 1 included one attention check item (for details, see Supplemental Material S1). After completing the prescreening, participants were informed about the purpose of the study and informed that they might receive an invitation to take part in Session 2 in a couple of days.

##### Session 2: main study

After providing informed consent, participants were told that they would be presented with the results of two scientific studies investigating the effectiveness of stricter regulations to decrease CO_2_ emissions (for detailed instructions, see online materials). Moreover, participants were told that the studies were conducted in two European countries (although the names of the two countries would not be disclosed for standardization purposes) – and that it will be their task to evaluate whether the studies support the conclusion that stricter regulations are an overall effective intervention or not. Whether participants first evaluated the pro-regulation study or the anti-regulation study was counterbalanced across participants. Which of the two sets of numbers was used for the pro-regulation study and for the anti-regulation study was counterbalanced across participants. The two counterbalancing factors did not influence the results, that is, there were no order effects, neither in Study 1 nor in Study 2 (see the Supplemental Material S7 and S18). After evaluating the two studies, participants responded to a multiple-choice attention check item (for details, see Supplemental Material S1). Participants were debriefed after completing Session 2. More specifically, participants were informed that the two studies were fictitious and that the existing scientific evidence indicates that stricter regulations can be considered a powerful means to reduce CO_2_ emissions.

### Results

#### Study evaluation

Overall, study evaluation accuracy rates were rather low, confirming the validity of our stimulus material as posing difficult statistical problems: 50.00% of the participants evaluated the pro-regulation study correctly (i.e., selected 4, 5, or 6 on the 6-point Likert scale), and 58.09% of the participants evaluated the anti-regulation study correctly (i.e., selected 1, 2, or 3). That is, the anti-regulation study was significantly more likely to be evaluated correctly than the pro-regulation study, *t*(555) = 2.77, *p* = 0.006, *d* = 0.12.

There was no significant difference between the time participants spent on evaluating the results of the pro-regulation study (*M*_*pro*_ = 117.08 s, *SD* = 121.84, Mdn. = 93 s) and the results of the anti-regulation study (*M*_*anti*_ = 110.88 s, *SD* = 81.04, Mdn. = 90 s), *t*(555) = 1.11, *p* = 0.266, *d* = 0.05. The fact that participants spent clearly more than one minute on average on evaluating the results indicates that participants engaged with the material and took the task seriously.

#### Biased reasoning and zero-order correlations

Attitude towards stricter regulations to reduce CO_2_ emissions was significantly correlated with the directional bias, indicating that participants showed attitude-consistent judgment (i.e., engaged in motivated reasoning) in the context of climate change, *r* = 0.09, *p* = 0.037. Zero-order correlations between variables are displayed in Table 1.

**Table 1:** Descriptive statistics and zero-order-correlations.

Variable	M	SD	1	2	3	4	5	6	7	8	9
1. Attitude	4.53	1.72	–								
2. Directional bias	− 0.24	2.08	0.09*	–							
3. Absolute bias	1.50	1.47	− 0.02	− 0.16**	–						
4. Numeracy	5.69	1.60	0.11*	− 0.09*	− 0.12**	–					
5. Need for Cognition	5.56	1.34	0.16**	− 0.01	− 0.02	0.23**	–				
6. Dark Factor	1.80	0.48	− 0.14**	− 0.05	− 0.01	.001	− 0.08	–			
7. Political orientation	3.48	1.33	− 0.33**	− 0.06	0.02	− 0.05	− 0.02	0.20**			
8. Attitude strength	4.55	1.20	0.63**	0.09*	− 0.03	0.07	0.18**	− 0.10*	− 0.24**		
9. Correct evaluation pro- study	0.50	0.50	0.05	0.62**	− 0.16**	0.13**	0.16**	− 0.08	− 0.06	0.10*	
10. Correct evaluation anti-study	0.58	0.49	− 0.04	− 0.60**	0.03	0.23**	0.14**	− 0.01	0.04	− 0.01	0.04

*p < 0.05, **p < 0.01; scale range of the variables: attitude: 1 to 7; directional bias: − 5 to 5; absolute bias: 0 to 5; numeracy: 0 to 8; Need for Cognition: 1 to 9; Dark Factor: 1 to 5; political orientation: 1 (extremely left) to 7 (extremely right); attitude strength: 1 to 7; evaluation: incorrect (0) or correct (1).

#### Potential factors related to biased reasoning

To investigate our research questions, we ran separate hierarchical regression analyses for each individual difference variable (i.e., numeracy, NFC, D). Note that we decided to run separate hierarchical regression analyses because we were mainly interested in the individual and separate contribution of each of the three factors. The predictors in each regression analysis were the attitude towards stricter regulations to reduce CO_2_ emissions (z-standardized, Step 1), the respective individual difference variable (z-standardized, Step 2), and the product term of the two variables (entered in Step 3). To check whether the results for each individual difference variable remain the same when the influence of the other predictors is controlled, we ran further hierarchical regression analyses in which we entered the other two individual difference variables as predictors in the first step. This did not change the results in meaningful ways (see Supplemental Material S9).

We ran separate regression analyses for the directional bias and the absolute bias as the criterion. The regression analyses using directional bias as the criterion help to determine whether the degree of attitude-consistent judgment (i.e., motivated reasoning) is moderated by the above-mentioned factors. In these regression analyses, the key question is whether the relationship between attitude and directional bias is moderated by the additional factor under investigation (i.e., whether the interaction effect is significant). The regression analyses using absolute bias as the criterion help to determine whether the degree of bias (irrespective of the direction) is predicted by the above-mentioned factors. In these regression analyses, the key question is whether the additional factor under investigation has an effect on the degree of absolute bias (i.e., whether the main effect is significant). That is, although we apply the same analytic strategy for both criteria in order to make the results easily comparable, the effect of interest differs depending on the used criterion. Tables for all regression analyses can be found in the Supplemental Material S9. Finally, we ran logistic regression analyses on study evaluation accuracy to investigate whether the likelihood of correct study interpretation is moderated by the above-mentioned factors. The results are presented in the Supplemental Material S10.

##### Directional bias

Attitude positively predicted the directional bias score, *B* = 0.18, *SEB* = 0.09, *p* = 0.037, *R*^*2*^ = 0.01. Numeracy was negatively related to the directional bias score, *B* = − 0.21, SEB = 0.09, *p* = 0.018, Δ*R*^*2*^ = 0.01. The interaction between attitude and numeracy was not significant, *B* = 0.15, SEB = 0.08, *p* = 0.073, ∆R^2^ = 0.006. In the next regression analysis, Need for Cognition was entered in the second step, yielding no significant association, *B* = -0.05, *SEB* = 0.09, *p* = 0.585, Δ*R*^*2*^ = 0.001. The interaction between attitude and Need for Cognition did not explain additional variance, *B* = 0.04, *SEB* = 0.08, *p* = 0.612, Δ*R*^*2*^ < 0.001. A similar pattern of results was found for the Dark Factor. It neither predicted the bias score, *B* = − 0.08, *SEB* = 0.09, *p* = 0.394, Δ*R*^*2*^ = 0.001, nor was an interaction present, *B* = 0.02, *SEB* = 0.09, *p* = 0.777, Δ*R*^*2*^ < 0.001. To check for higher-order interactions involving attitude as one of the predictors, we ran an additional regression analysis with all variables and the product term of these variables as predictors. No three-way interaction was observed that involved attitude as one of the predictors, all *p*s > 0.249 (for details, see the Supplemental Material S9).

##### Absolute bias

Attitude towards stricter regulations was unrelated to the absolute bias score, *B* = − 0.03, *SEB* = 0.06, *p* = 0.614, *R*^*2*^ < 0.001. Numeracy, entered as an additional predictor in the second step, was negatively related to the absolute bias score, *B* = − 0.17, SEB = 0.06, *p* = 0.007, Δ*R*^*2*^ = 0.01. The interaction between attitude and numeracy was not significant, *B* = − 0.06, SEB = 0.06, *p* = 0.313, ∆R^2^ = 0.002. In our second analysis, Need for Cognition did not predict the absolute bias score, *B* = − 0.03, *SEB* = 0.06, *p* = 0.653, *R*^*2*^ < 0.001, but the interaction between attitude and Need for Cognition was significant, *B* = − 0.12, *SEB* = 0.06, *p* = 0.043, ∆R^2^ = 0.007. The Dark Factor was unrelated to the absolute bias score, *B* = − 0.02, SEB = 0.06, *p* = 0.775, Δ*R*^*2*^ < 0.001, and no interaction between attitude and the Dark Factor was observed, *B* = 0.08, SEB = 0.06, *p* = 0.212, ∆R^2^ = 0.003. To check for higher-order interactions involving attitude as one of the predictors, we ran an additional regression analysis with all variables and the product term of these variables as predictors. We found no indication for the presence of three-way interactions that involved attitudes, all *p*s > 0.546 (for details, see the Supplemental Material S9).

##### Bayes regressions

**Figure 4:**
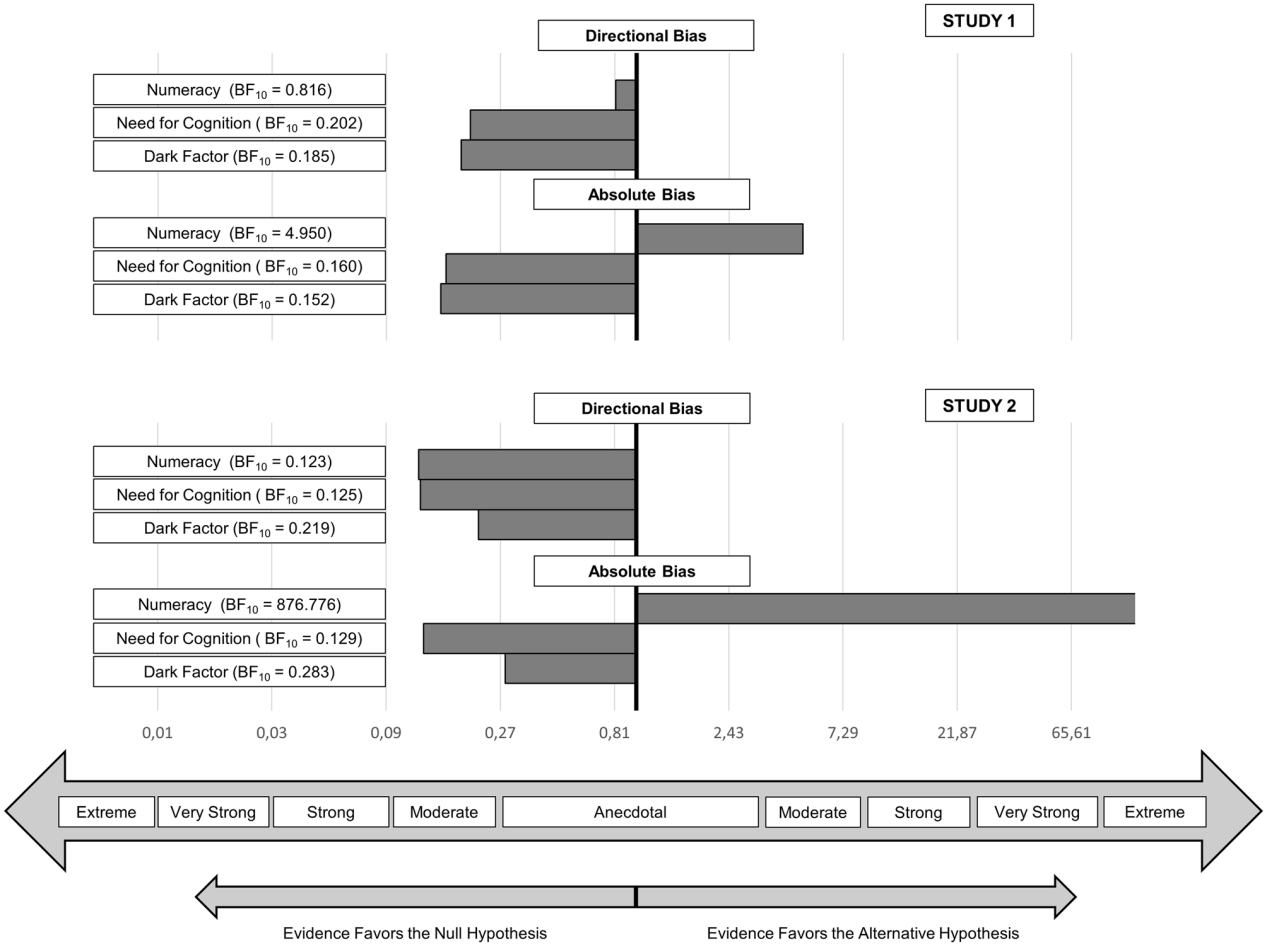
Bayes Linear Regression Results. Results of Bayes Regression Analyses with JASP default priors. For results on the directional bias as the criterion, BF10 for the model with the interaction between attitude and the focal individual difference variable versus the null model that includes the two main effects is plotted. For results on the absolute bias as the criterion, BF10 for the model with the focal individual difference variable versus the null model that includes attitude is plotted. For BF10 < 1, evidence favors the null hypothesis, for BF10 > 1, evidence favors the alternative hypothesis. Descriptive labels regarding the strength of evidence follow extant recommendations^88,89^.

We completed our analyses with Bayes linear regression analyses as they allow for an interpretation of the degree of evidence favoring the null hypothesis^76–78^. Of particular interest regarding the directional bias were comparisons between the interactions between attitude and the focal individual difference variable versus the null model. The results (see Fig. 4) indicate moderate support for the null hypothesis in models with the Need for Cognition or the Dark Factor. Results were inconclusive for Numeracy. Of particular interest regarding the absolute bias were comparisons between the main effects of the focal individual difference variable and the null model. These analyses indicate moderate support for the null hypothesis in models with the Need for Cognition or the Dark Factor, as well as moderate support for the alternative hypothesis that numeracy is negatively linked to the absolute bias.

### Discussion

Study 1 revealed three important findings. First, participants engaged in motivated reasoning in the context of climate change, confirming the results from previous studies^20–29^. The second important finding concerns the role of numeracy. On the one hand, we found that higher numeracy was associated with a reduced absolute bias (i.e., a reduced degree of asymmetric reasoning), indicating that more numerate individuals were better at differentiating the outcome of the two fictitious studies. This result is in line with previous research using the absolute bias score as a dependent variable in a different context^32^. On the other hand, numeracy did not significantly moderate the relationship between attitude and directional bias. Note, however, that this nonsignificant finding was inconclusive as indicated by the Bayes Factors, making definitive interpretations difficult. Third, the other factors investigated in the present study (i.e., Need for Cognition, Dark Factor of Personality) did not influence the participants’ performance. More specifically, none of the two factors moderated the relationship between attitude and directional bias (i.e., the degree of attitude-consistent judgment) and none of the two factors had an influence on the degree of the absolute bias (i.e., the degree of asymmetric reasoning regarding the two fictitious studies). The finding that Need for Cognition moderated the relationship between attitude and absolute bias was not part of our main research questions and will therefore not considered further in the following. In order to shed more light on the role of numeracy and to confirm the observation that none of the other two factors seem to play a major role for motivated reasoning in the context of climate change, we decided to run a preregistered conceptual replication study with a larger sample.

## Study 2

### Method

#### Participants

We aimed for a final sample of at least twice the size in the main study (i.e., at least 1100 participants). The study was preregistered (10.17605/osf.io/94fn3). Participants were recruited via Prolific (www.prolific.com). Participants provided informed consent. All data exclusions, manipulations, and measures are reported. The study was programmed on SoSciSurvey^69^.

##### Session 1: prescreening

In Session 1, participants were prescreened for their attitude towards stricter regulations to reduce CO_2_ emissions. In total, 1604 participants completed the prescreening. Participation was only possible for Prolific users with an approval rate of at least 98% who were fluent in English and currently living in the United Kingdom. Participants who had already participated in Study 1 were not eligible for participation in Study 2. Participants were compensated with £1.65. Session 1 lasted about ten minutes. Data collection took place on January 11, 2023.

##### Session 2: main study

To account for exclusions and potential dropout between sessions, we decided to invite 1450 participants for Session 2. An equal distribution of participants across the seven scale points of the regulation attitude item would have implied inviting 207 participants per scale point for Session 2. Whenever we had 207 or fewer participants on one scale point of the regulation attitude item, all of these participants were invited for Session 2; whenever we had more than 207 participants on one of the seven points of the scale, we randomly drew a subset of participants (for more information about the participants who were selected for participation in Session 2 see the Supplemental Material S13 and S14).

From the total of 1450 participants that were invited to participate in Session 2, 1371 participants completed the study. Participants who did not pass all attention checks (*N* = 160) were excluded from the analysis. We also excluded participants who had noticeable short (i.e., less than one third of the median response time; *N* = 7) or long (i.e., more than six times the median response time; *N* = 6) response times, indicating careless responding. Thus, the final sample consisted of 1198 participants (*M*_*age*_ = 41.34, *SD* = 13.39, 18–81 years; 550 male, 637 female, 8 other, 3 prefer not to say; see Supplemental Material S15–S17 for information about political orientation, ethnicity, and education). Participants were compensated with £1.05 upon study completion. Session 2 lasted about six minutes. Data collection took place from January 22 to January 25, 2023.

#### Materials and procedure

As Study 2 was designed to be a conceptual replication study, we closely followed the design and procedure of Study 1. As the correlation between attitude and directional bias was quite low in Study 1 (*r* = 0.09) and as the number of participants who opposed stricter regulations was clearly lower than the number of participants who supported stricter regulations, we implemented three changes hoping to strengthen the motivated reasoning effect and to achieve a slightly more balanced sample regarding attitude. First, the attitude item was changed from “Governments should enforce stricter regulations to decrease CO_2_ emissions even if these regulations restrict the freedom of the individual” to “Governments should enforce stricter regulations to decrease CO_2_ emissions, which restrict the freedom of the individual”. Second, we changed the labels on the two-by-two contingency tables from “Cities/Districts with stricter regulations” versus “Cities/Districts without stricter regulations” (see Fig. 2) to “Cities/Districts with regulations restricting the freedom of the individual” versus “Cities/Districts without regulations restricting the freedom of the individual”. Third, the prescreening (Session 1) only included the attitude item but not the six items measuring attitude strength; the attitude item was presented again and this time together with the six items measuring attitude strength in the beginning of Session 2 directly before evaluating the two fictitious studies. A fourth change was implemented in order to allow additional exploratory analyses (for results, see the Supplemental Material S22): Below each of the two fictitious studies, we added two more items, one item measuring how confident participants are that their response to the fictitious study was correct (“How confident are you that your answer is correct?”; 6-point Likert scale ranging from 1 (very unconfident) to 6 (very confident); see^79^) and one item measuring how trustworthy participants find the study’s result (“How trustworthy do you think the results of this study are?”; 6-point Likert scale ranging from 1 (not at all trustworthy) to 6 (very trustworthy); see^80^).

The rest of the study materials as well as study procedure were identical to Study 1 (for the attention check items, see the Supplemental Material S12). The answers on the attitude items in Session 1 and Session 2 were highly correlated, *r* = 0.744, *p* < 0.001. The statistical analyses were conducted with the answers on the attitude item from Session 2. The internal consistency of the 6-item attitude strength scale was good, α = 0.85. The internal consistency of the NFC scale was excellent, α = 0.94. The internal consistency of the scale measuring D was good, α = 0.86.

### Results

#### Study evaluation

Overall, study evaluation accuracy rates were rather low, confirming the validity of our stimulus material as posing difficult statistical problems: 43.82% of the participants evaluated the pro-regulation study correctly (i.e., selected 4, 5, or 6 on the 6-point Likert scale), and 57.10% of the participants evaluated the anti-regulation study correctly (i.e., selected 1, 2, or 3). That is, the anti-regulation study was significantly more likely to be evaluated correctly than the pro-regulation study, *t*(1197) = -6.67, *p* < 0.001, *d* = 0.19.

There was no significant difference between the time participants spent on evaluating the results of the pro-regulation study (*M*_*pro*_ = 87.83 s, *SD* = 67.79, Mdn. = 73 s) and the results of the anti-regulation study (*M*_*anti*_ = 93.54 s, *SD* = 88.58, Mdn. = 73 s), *t*(1197) =− 1.89, *p* = 0.059, *d* = 0.05. The fact that participants spent more than a minute on average on evaluating the results indicates that participants engaged with the material and took the task seriously.

#### Biased reasoning and zero-order correlations

Attitude towards stricter regulations to reduce CO_2_ emissions was significantly correlated with the directional bias, indicating that participants showed attitude-consistent judgment (i.e., engaged in motivated reasoning) in the context of climate change, *r* = 0.11, *p* < 0.001. Zero-order correlations between variables are displayed in Table 2.

**Table 2:** Descriptive statistics and zero-order-correlations.

Variable	M	SD	1	2	3	4	5	6	7	8	9
1. Attitude	4.35	1.64	–								
2. Directional bias	− 0.46	2.10	0.11**	–							
3. Absolute bias	1.56	1.47	− 0.07*	− 0.29**	–						
4. Numeracy	5.70	1.53	0.08**	− 0.02	− 0.13**	–					
5. Need for Cognition	5.67	1.32	0.14**	0.02	− 0.03	0.23**	–				
6. Dark Factor	1.80	0.50	− 0.11**	0.02	− 0.03	0.04	− 0.14**	–			
7. Political orientation	3.35	1.31	− 0.30**	− 0.02	0.07*	− 0.09**	− 0.05	0.19**			
8. Attitude strength	4.61	1.13	0.48**	0.01	− 0.01	0.02	0.23**	− 0.16**	− 0.21**		
9. Correct evaluation pro-study	0.44	0.50	0.11**	0.64**	− 0.20**	0.14**	0.06*	0.01	− 0.07*	− 0.02	
10. Correct evaluation anti-study	0.57	0.50	0.02	− 0.58**	0.12**	0.20**	0.04	0.01	− 0.05	0.01	0.04

*p < 0.05, **p < 0.01; scale range of the variables: attitude: 1 to 7; directional bias: − 5 to 5; absolute bias: 0 to 5; numeracy: 0 to 8; Need for Cognition: 1 to 9; Dark Factor: 1 to 5; political orientation: 1 (extremely left) to 7 (extremely right); attitude strength: 1 to 7; evaluation: incorrect (0) or correct (1).

#### Potential factors related to biased reasoning

As in Study 1, we ran separate hierarchical regression analyses for each individual difference variable (i.e., numeracy, NFC, D). Again, we also ran further hierarchical regression analyses in which we entered the other two individual difference variables as predictors in the first step to check whether the results for each individual difference variable remain the same when the influence of the other predictors is controlled. Again, this did not change the results in meaningful ways (see Supplemental Material S20). Note that the preregistration stated that we would run one-step regression analyses with attitude, one of the three factors potentially influencing motivated reasoning (i.e., numeracy, NFC, D), and the product term of the two variables as predictors and bias as the criterion. However, we decided that conducting hierarchical regression analyses would be easier to report and understand. Note that the results do not change when running the preregistered one-step analyses instead of the hierarchical regression analyses. Tables for all regression analyses can be found in the Supplemental Material S20. Finally, we ran logistic regression analyses on study evaluation accuracy. The results are presented in the Supplemental Material S21.

##### Directional bias

Again, attitude positively predicted the directional bias score, *B* = 0.23, *SEB* = 0.06, *p* =  < 0.001, *R*^*2*^ = 0.01. Numeracy did not predict the directional bias score, *B* =− 0.06, SEB = 0.06, *p* = 0.311, Δ*R*^*2*^ = 0.001, and no interaction between attitude and numeracy was observed, *B* = 0.01, SEB = 0.06, *p* = 0.907, ∆R^2^ < 0.001. In the subsequent regression analysis, Need for Cognition was entered in the second step, yielding no significant association, *B* = 0.01, *SEB* = 0.06, *p* = 0.908, Δ*R*^*2*^ < 0.001. The interaction between attitude and Need for Cognition did not explain additional variance, *B* = − 0.01, *SEB* = 0.06, *p* = 0.832, Δ*R*^*2*^ < 0.001. Likewise, the Dark Factor did neither predict the bias score, *B* = 0.07, *SEB* = 0.06, *p* = 0.233, Δ*R*^*2*^ = 0.001, nor was an interaction present, *B* = 0.06, *SEB* = 0.06, *p* = 0.275, Δ*R*^*2*^ = 0.001. To check for higher-order interactions involving attitude as one of the predictors, we ran an additional regression analysis with all variables and the product term of these variables as predictors. No three-way interactions that involved attitudes were observed, all *p*s > 0.159 (for details, see the Supplemental Material S20).

##### Absolute bias

Attitude negatively predicted the absolute bias score, *B* = -0.11, *SEB* = 0.04, *p* = 0.011, *R*^*2*^ = 0.005. When numeracy was entered as an additional predictor in the second step, higher numeracy predicted a reduced absolute bias score, *B* = -0.18, SEB = 0.04, *p* < 0.001, Δ*R*^*2*^ = 0.02. The interaction between attitude and numeracy was not significant, *B* = − 0.07, SEB = 0.04, *p* = 0.091, ∆R^2^ = 0.002. Our second analysis involved the Need for Cognition, which did not predict the absolute bias score when entered in the second step, *B* = -0.03, *SEB* = 0.04, *p* = 0.461, *R*^*2*^ < 0.001, and the interaction between attitude and Need for Cognition was not significant, *B* = − 0.04, *SEB* = 0.04, *p* = 0.337, ∆R^2^ = 0.001. The Dark Factor was unrelated to the absolute bias score, *B* = − 0.06, SEB = 0.04, *p* = 0.144, Δ*R*^*2*^ = 0.002, and no interaction between attitude and the Dark Factor was observed, *B* =  < 0.01, SEB = 0.04, *p* = 0.986, ∆R^2^ < 0.001. To check for higher-order interactions involving attitude as one of the predictors, we ran an additional regression analysis with all variables and the product term of these variables as predictors. Again, none of the three-way interactions that involved attitudes as a predictor yielded a significant higher-order effect, all *p*s > 0.270 (for details, see the Supplemental Material S20).

##### Bayes regressions

We again completed our analyses with Bayes linear regression analyses to gain additional insights regarding the degree of evidence favoring the null hypothesis^76–78^. Bayes analyses were first conducted with the directional bias score as the criterion and the interactions between attitude and the individual difference variable vs. the null model as the effect of interest. For all three individual difference variables the results indicate moderate support for the null hypothesis (see Fig. 4). Next, analyses were conducted with the absolute bias score as the criterion and the association with the individual difference variable vs. the null model as the effect of interest. These analyses indicate moderate support for the null hypothesis in models with the Need for Cognition or the Dark Factor the predictor, as well as very strong support for the hypothesis that numeracy is negatively linked to the absolute bias.

## General discussion

The present research had two main goals. The first goal was to investigate whether individuals engage in motivated reasoning in the context of climate change using a design that accounts for the limitations of previous studies. In order to allow a nuanced analysis of motivated reasoning, we calculated two biases, a directional and an absolute bias. The second goal was to examine potential moderating factors of motivated reasoning. More specifically, we sought to investigate the influence of the ability to perform the numerical-statistical task at hand (operationalized in terms of numeracy), the willingness to use this ability (operationalized in terms of Need for Cognition), and the stable tendency to maximize one’s individual utility (operationalized in terms of the Dark Factor of Personality).

Regarding the first goal, we found clear evidence across two studies that participants engaged in motivated reasoning when evaluating studies on the effectiveness of stricter regulations to reduce CO_2_ emissions. In particular, the more strongly participants opposed stricter regulations, the more strongly they exhibited a directional bias towards interpreting the studies as showing that stricter regulations are counterproductive. Conversely, the more strongly participants favored stricter regulations, the more strongly they exhibited a directional bias towards interpreting the studies as showing that stricter regulations are effective. These results are in line with the previous studies on motivated reasoning in the context of climate change^20–29^. Importantly, the present research does not only confirm these results but also extends the existing body of knowledge in several ways: We demonstrated motivated reasoning in the context of climate change when evaluating scientific evidence on the effectiveness of specific policies to reduce CO_2_ emissions instead of merely addressing the question whether climate change is anthropogenic. Thus, motivated reasoning also extends to more nuanced discourses about the pros and cons of specific policies. Furthermore, we demonstrated motivated reasoning when directly measuring participants’ attitude on stricter regulations instead of using political orientation as a proxy. Thus, motivated reasoning is not limited to highly identity-relevant social categories such as political orientation. With respect to the latter, note that political orientation was – as indicated by the zero-order correlations (see Table 1 and Table 2) – not associated with directional bias, suggesting that it is important to address the attitudes underlying motivated reasoning rather than using broad identity categories, at least when investigating a topic that is less polarized along party lines^9,81^.

Regarding our findings for motivated reasoning, two more aspects deserve attention. On the one hand, and as pointed out in the introduction, motivated reasoning is usually seen as a combination of the motivated acceptance of attitude-consistent information and the motivated rejection of attitude-inconsistent information. Accordingly, our directional bias score combines the participants’ responses to attitude-consistent and attitude-inconsistent information in a joint estimate. Nevertheless, future research using different designs and bias estimates could strive to disentangle the processes of motivated acceptance and motivated rejection and their contribution to biased reasoning. On the other hand, the correlation between attitude and the directional bias score was robust but relatively small (about 0.10 in both studies), suggesting that motivated reasoning effects in the context of evaluating policies to reduce CO_2_ emissions exist but might be somewhat limited in size. Interestingly, a study on motivated reasoning in the context of mask mandates during the COVID-19 pandemic, which used the same design as the present studies, found a substantially higher correlation between attitude and directional bias of 0.34^32^. This indicates that the size of motivated reasoning effects may vary depending on contextual factors such as the topic under investigation. Note, however, that even relatively small effects as observed in the present studies can be of great practical importance especially when they apply to large groups of individuals^82^. Simply put, as motivated reasoning is usually assumed to be a general phenomenon, its societal impact can be large even if the effect itself is small.

Regarding the second goal, first note that the low correlations between the three dimensions of individual differences (see Tables 1 and 2) confirm our idea that these dimensions are indeed relatively distinct from one another. As far as the influence of these three variables on motivated reasoning is concerned, the results across our two studies are remarkably similar: In short, only numeracy was clearly associated with the degree of biased reasoning in the sense that higher numeracy predicted a reduced absolute bias. Neither NFC nor D were related to the directional or the absolute bias.

The finding that higher numeracy is associated with a reduced absolute bias is in line with previous research demonstrating that numeracy can lead to more objective reasoning and better decision-making even in the context of highly politicized issues^32,47,48^. At the same time, the finding that numeracy did not moderate the relationship between attitude and directional bias contradicts those studies, which have found more pronounced motivated reasoning among highly numerate individuals^21,25,26,42,43^. Although the latter studies have been criticized on methodological and theoretical grounds^44^, further investigating under which circumstances numeracy is associated with reduced and under which circumstances it is associated with exacerbated motivated reasoning is an important topic for future research. Note that the mechanisms potentially underlying the contradictory findings (i.e., using numerical-statistical abilities to arrive at a more objective evaluation of evidence versus using these abilities to rationalize prior beliefs) are not mutually exclusive; hence, which mechanism is more dominant may depend on various contextual factors and boundary conditions. Against this background, the present research also underlines the importance of a nuanced analysis of motivated reasoning: Rather than investigating motivated reasoning as a single construct, it may be advisable to look at different biases that elucidate different aspects of motivated reasoning, such as the directional and the absolute bias used in the present study. In this respect, our results suggest that numeracy is not associated with the degree of attitude-consistent reasoning but with a better ability to understand that the two fictitious studies show different outcomes.

Given that we have found consistent evidence that higher numeracy is associated with reduced biased reasoning, it appears essential to foster statistical education in the general population^83,84^: If individuals lack the necessary abilities to understand the scientific evidence that is being discussed in the public discourse and that underlies policy decisions, they potentially also lack the ability to act as autonomous and responsible citizens who do their part to address global challenges such as climate change. In other words, our findings point to the importance of systemic reforms that provide an environment in which individuals can develop the skills that are needed in the complex modern world: While motivated reasoning may be a universal cognitive mechanism, the degree to which individuals correctly interpret scientific evidence will crucially depend on their access to appropriate educational environments. In the short run, it also seems important to support numerical information with visualizations and explanations that are easy to understand and that can help to bridge existing knowledge gaps^85,86^.

The encouraging result that higher numeracy is associated with a reduced absolute bias is further underlined by the observation that degree of motivated reasoning does not vary as a function of the individuals’ NFC or D, that is, as a function of two individual difference variables, which would be much harder to change. With respect to NFC, our results are in line with a previous study which also found no significant effects^20^. Thus, the present research lends credibility to the idea that the positive effects of a high NFC (i.e., being more likely to be driven by accuracy goals)^53^ might be counterbalanced by the negative effects of a high NFC (i.e., the tendency to have stronger and less ambivalent attitudes that are more resistant to change)^54,55^. With respect to D, one may speculate that the problematic epistemic beliefs which are fueled by a pronounced Dark Factor^60–62^ lead to cognitive processes that are conceptually distinct from motivated reasoning. More specifically, one could assume that individuals high in D are skeptical about scientific evidence *in general* because they believe, for instance, that truth is political and ultimately a matter of power, while motivated reasoning refers to biased information processing when being confronted with *specific* scientific evidence. However, further investigating these potential differences could be another important topic for future research.

Three aspects of the stimulus material need emphasis. First, evaluation accuracy was relatively low across both studies: Only about half of the participants indicated correctly whether the contingency tables showed that stricter regulations are effective or counterproductive. This demonstrates that analyzing contingency tables indeed poses a difficult statistical problem to individuals and that the correct interpretation of the provided data was not obvious. As real scientific evidence that individuals might encounter when looking for information about climate change is likely to be difficult to understand, the stimulus material offers a valid test for motivated reasoning. At the same time, it should not be forgotten that understanding data on climate change arguably requires more sophisticated statistical knowledge than the ability to compare the ratios of contingency tables – making the participants’ poor performance even more worrying and providing more evidence for our claim that improving statistical literacy in the general population is highly desirable.

Second, we chose to present the fictitious studies in the form of two-by-two contingency tables because this allowed a well-controlled presentation of the data, ensuring that the relevant information is easily available to participants. In addition, this also helped to make certain that participants’ responses were based on reasoning processes rather than their ability to read and understand texts or their general world knowledge. That being said, we want to emphasize that studies on motivated reasoning should be conducted using different materials to ensure the generalizability of the findings. Importantly, this particularly applies to investigations concerning the factors influencing motivated reasoning: While there is already ample evidence regarding the existence of motivated reasoning in the context of climate change using different kinds of stimulus material^20–29^, this is not the case for all factors investigated in the present studies. Hence, we encourage independent replications of our findings using different materials.

Third, in both Study 1 and Study 2 the percentage of participants who evaluated the anti-regulation study correctly was higher than the percentage of participants who evaluated the pro-regulation study correctly. As research clearly indicates that enforcing stricter regulations can be a powerful means to decrease CO_2_ emissions^87^, one might assume that participants expected to be presented with evidence supporting the effectiveness of stricter regulations. In case that—at least some—participants based their response on such a prior expectation, however, this would result in exactly the opposite pattern, as a superficial evaluation of the anti-regulation study suggests that stricter regulations are effective, while a superficial evaluation of the pro-regulation study suggests that stricter regulations are counterproductive. That is, in this case, the participants’ performance in the pro-regulation study should have been better than the performance in the anti-regulation study. A possible explanation for the observed pattern could be that the participants’ expectations were not so much shaped by the existing scientific evidence but rather by societal debates, which often question how much of a difference stricter regulations on a local level can actually make when climate change is a global problem. However, this finding deserves further investigation.

In sum, the present research offers good and bad news. The bad news is that motivated reasoning poses a challenge to modern societies facing climate change. Importantly, this does not only apply to the general question whether climate change is anthropogenic, but also to the evaluation of specific scientific evidence. The good news is that the degree to which individuals engage in biased reasoning mainly depends on the individuals’ abilities (such as numeracy), which can in principle be improved through education, and not so much on rather stable personality characteristics (such as the Dark Factor of Personality) or thinking styles (such as Need for Cognition), which are arguably harder to change.

### Supplementary Information

Supplementary Information.

## Data Availability

Data and analysis code for both Study 1 and Study 2 can be downloaded at 10.17605/osf.io/nvek2.
