# Supplementary material for: Motivated reasoning about climate change and the influence of Numeracy, Need for Cognition, and the Dark Factor of Personality
Source: Sci Rep. 2024 Mar 7;14:5615. doi: 10.1038/s41598-024-55930-9 (PMC10920913; doi:10.1038/s41598-024-55930-9)
Supplement: Supplementary file 1 — Supplementary Information. [file 41598_2024_55930_MOESM1_ESM.docx]

**SUPPLEMENTAL MATERIAL**

**Motivated Reasoning about Climate Change and the Influence of Numeracy, Need for Cognition, and the Dark Factor of Personality**

**STUDY 1**

S1 Attention check items

S2 Selection of participants for the Main Study (Session 2)

S3 Distribution of the participants with respect to their attitude towards stricter regulations to reduce CO_2_ emissions in the final sample (*N* = 556)

S4 Ethnicity

S5 Education

S6 Political Orientation

S7 Analysis of potential order effects

S8 Results for attitude strength

S9 Additional tables for regression analyses

S10 Predictors of study evaluation accuracy

S11 Additional information about the stimulus material

**STUDY 2**

S12 Attention check items

S13 Selection of participants for the Main Study (Session 2)

S14 Distribution of the participants with respect to their attitude towards stricter regulations to reduce CO_2_ emissions in the final sample (N = 1198)

S15 Ethnicity

S16 Education

S17 Political Orientation

S18 Analysis of potential order effects

S19 Results for attitude strength

S20 Additional tables for regression analyses

S21 Predictors of study evaluation accuracy

S22 Trustworthiness of study results and confidence in one’s interpretation

**STUDY 1**

**S1 Attention check items**

In Session 1, participants answered one attention check item (“It’s important that you pay attention to this study. Please tick ‘extremely important’”) that was intermixed with the items regarding the attitude towards stricter regulations to reduce CO_2_ emissions. In Session 2, participants answered one multiple choice attention check item after evaluating the two fictitious studies in order to ensure that the participants had read the instructions carefully (“The two studies on the effects of stricter regulations to reduce CO_2_ emissions were conducted: (a) in two states of the US: The names of the states were not discloses; (b) in two states of the US: Ohio and Michigan; (c) in two European countries: France and Germany; (d) in two European countries: The names of the countries were not disclosed”).

**S2 Selection of participants for the Main Study (Session 2)**

From the 902 participants who completed Session 1, 721 participants were invited for participation in Session 2. We decided not to invite participants for Session 2 who had failed the attention check (see below; *N* = 8) or who had noticeable short (i.e., less than one third of the median response time; *N* = 2) or long (i.e., more than six times the median response time; *N* = 0) response times indicating careless responding as well as one participant who had complained about the length of the study. The subset of 721 participants was drawn from the remaining participants following the procedure described in the main text.

**S3 Distribution of the participants with respect to their attitude towards stricter regulations to reduce CO_2_ emissions in the final sample (*N* = 556)**

| **Attitude towards stricter regulations to reduce CO_2_ emissions** | **Number of participants** |
| --- | --- |
| 1 = extremely oppose | 30 |
| 2 | 47 |
| 3 | 88 |
| 4 | 88 |
| 5 | 117 |
| 6 | 110 |
| 7 = extremely favor | 76 |

**S4 Ethnicity**

| **Ethnicity** | **Number of participants** |
| --- | --- |
| White | 494 |
| Black/Black British | 14 |
| Asian/Asian British | 27 |
| Mixed | 16 |
| Other | 2 |
| Prefer not to say | 3 |

**S5 Education**

| **Education** | **Number of participants** |
| --- | --- |
| Primary school | 2 |
| Secondary school up to 16 years | 48 |
| Higher or secondary or further education (A-levels, BTEC, etc.) | 105 |
| College or university | 266 |
| Post-graduate degree | 132 |
| Other | 1 |
| Prefer not to say | 2 |

**S6 Political Orientation**

| **Political orientation** | **Number of participants** |
| --- | --- |
| Extremely left | 23 |
| Left | 130 |
| Center left | 130 |
| Center | 148 |
| Center right | 81 |
| Right | 41 |
| Extremely right | 3 |

**S7 Analysis of potential order effects**

*Means and Standard Deviations (in parentheses) of Directional Bias and Absolute Bias as a Function of Study Order (Pro-Regulation Study first, Anti-Regulation Study second vs. Anti-Regulation Study first, Pro-Regulation Study second) and Study Number Mapping (Pro-Regulation Study with N_cities/districts_ = 426, Anti-Regulation Study with N_cities/districts_ = 390 vs. Pro-Regulation Study with N_cities/districts_ = 390, Anti-Regulation Study with N_cities/districts_ = 426)*

|  | *N* | Directional Bias | Absolute Bias |
| --- | --- | --- | --- |
| Study Order: Pro-Regulation Study first, Anti-Regulation Study second |  |  |  |
| Study Number Mapping: |  |  |  |
| Pro-Regulation Study with *N* = 426,  Anti-Regulation Study with *N* = 390 | 137 | -0.57 (2.10) | 1.52 (1.55) |
| Pro-Regulation Study with *N* = 390,  Anti-Regulation Study with *N* = 426 | 145 | -0.20 (1.96) | 1.40 (1.38) |
| Study Order: Anti-Regulation Study first, Pro-Regulation Study second |  |  |  |
| Study Number Mapping: |  |  |  |
| Pro-Regulation Study with *N* = 426,  Anti-Regulation Study with *N* = 390 | 144 | -0.25 (2.14) | 1.51 (1.53) |
| Pro-Regulation Study with *N* = 390,  Anti-Regulation Study with *N* = 426 | 130 | 0.07 (2.05) | 1.58 (1.31) |

*Statistics from the univariate 2 (Study Order: Pro-Regulation Study first, Anti-Regulation Study second vs. Anti-Regulation Study first, Pro-Regulation Study second) x 2 (Study Number Mapping: Pro-Regulation Study with N_cities/districts_ = 426, Anti-Regulation Study with N_cities/districts_ = 390 vs. Pro-Regulation Study with N_cities/districts_ = 390, Anti-Regulation Study with N_cities/districts_ = 426) ANOVAs on Directional Bias and Absolute Bias.*

|  | Directional Bias | | | Absolute Bias | | |
| --- | --- | --- | --- | --- | --- | --- |
|  | *F*  (1, 552) | *p* | η_p_^2^ | *F*  (1, 552) | *p* | η_p_^2^ |
| Main Effect Study Order | 2.82 | .094 | .005 | 0.49 | .483 | .001 |
| Main Effect Study Number Mapping | 3.86 | .050 | .007 | 0.05 | .823 | <.001 |
| Interaction Study Order * Study Number Mapping | 0.02 | .886 | <.001 | 0.54 | .462 | .001 |

**S8 Results for attitude strength**

To investigate our research questions, we ran separate hierarchical regression analyses for each factor potentially influencing motivated reasoning. The predictors in each regression analysis were the attitude towards stricter regulations to reduce CO_2_ emissions (z-standardized, Step 1), the respective factor potentially influencing motivated reasoning (z-standardized, entered in Step 2), and the product term of the two variables (entered in Step 3). We ran separate regression analyses for the directional bias and the absolute bias as the criterion.

***Directional Bias***

Attitude strength as the second predictor in the equation yielded no significant effect, with *B* = .12, *SEB* = .11, *p* = .283, Δ*R^2^* = .002, for the association, and *B* = .12, *SEB* = .09, *p* = .185, Δ*R^2^* = .003, for the interaction.

***Absolute Bias***

Likewise, when absolute bias served as the criterion, attitude strength as the second predictor in the equation yielded no significant effect, with *B* = -.04, *SEB* = .08, *p* = .637, Δ*R^2^* < .001, for the association, and *B* = .03, *SEB* = .07, *p* = .608, Δ*R^2^* < .001 for the interaction.

**S9 Additional tables for regression analyses**

**Tables for regression analyses reported in the manuscript**

**a. Directional Bias**

*Numeracy*

|  | **Model 1** | | | **Model 2** | | | **Model 3** | | |
| --- | --- | --- | --- | --- | --- | --- | --- | --- | --- |
|  | *B* | *SEB* | *p* | *B* | *SEB* | *p* | *B* | *SEB* | *p* |
| Intercept | -.24 | .09 | .006 | -.24 | .09 | .006 | -.26 | .09 | .003 |
| Attitude | .18 | .09 | .037 | .21 | .09 | .019 | .22 | .09 | .013 |
| Numeracy |  |  |  | -.21 | .09 | .018 | -.21 | .09 | .019 |
| Attitude×Numeracy |  |  |  |  |  |  | .15 | .08 | .073 |
| R² |  | .01 |  |  | .02 |  |  | .02 |  |
| Adjusted R² |  | .01 |  |  | .01 |  |  | .02 |  |
| *F* for change in R² |  | 4.28 |  |  | 5.66 |  |  | 3.24 |  |

*Need for Cognition*

|  | **Model 1** | | | **Model 2** | | | **Model 3** | | |
| --- | --- | --- | --- | --- | --- | --- | --- | --- | --- |
|  | *B* | *SEB* | *p* | *B* | *SEB* | *p* | *B* | *SEB* | *p* |
| Intercept | -.24 | .09 | .006 | -.24 | .09 | .006 | -.25 | .09 | .005 |
| Attitude | .18 | .09 | .037 | .19 | .09 | .032 | .19 | .09 | .032 |
| NFC | -.24 | .09 | .006 | -.05 | .09 | .585 | -.05 | .09 | .598 |
| Attitude×NFC |  |  |  |  |  |  | .04 | .08 | .612 |
| R² |  | .01 |  |  | .01 |  |  | .01 |  |
| Adjusted R² |  | .01 |  |  | .01 |  |  | <.01 |  |
| *F* for change in R² |  | 4.38 |  |  | 0.30 |  |  | 0.26 |  |

*Dark Factor*

|  | **Model 1** | | | **Model 2** | | | **Model 3** | | |
| --- | --- | --- | --- | --- | --- | --- | --- | --- | --- |
|  | *B* | *SEB* | *p* | *B* | *SEB* | *p* | *B* | *SEB* | *p* |
| Intercept | -.24 | .09 | .006 | -.24 | .09 | .006 | -.24 | .09 | .007 |
| Attitude | .18 | .09 | .037 | .17 | .09 | .052 | .17 | .09 | .052 |
| Dark Factor |  |  |  | -.08 | .09 | .394 | -.08 | .09 | .395 |
| Attitude×Dark Factor |  |  |  |  |  |  | .02 | .08 | .777 |
| R² |  | .01 |  |  | .01 |  |  | .01 |  |
| Adjusted R² |  | .01 |  |  | .01 |  |  | <.01 |  |
| *F* for change in R² |  | 4.38 |  |  | 0.73 |  |  | 0.08 |  |

**b. Absolute Bias**

*Numeracy*

|  | **Model 1** | | | **Model 2** | | | **Model 3** | | |
| --- | --- | --- | --- | --- | --- | --- | --- | --- | --- |
|  | *B* | *SEB* | *p* | *B* | *SEB* | *p* | *B* | *SEB* | *p* |
| Intercept | 1.50 | .06 | <.001 | 1.50 | .06 | <.001 | 1.51 | .06 | <.001 |
| Attitude | -.03 | .06 | .614 | -.01 | .06 | .832 | -.02 | .06 | .765 |
| Numeracy |  |  |  | -.17 | .06 | .007 | -.17 | .06 | .007 |
| Attitude×Numeracy |  |  |  |  |  |  | -.06 | .06 | .313 |
| R² |  | <.01 |  |  | .01 |  |  | .02 |  |
| Adjusted R² |  | <.01 |  |  | .01 |  |  | .01 |  |
| *F* for change in R² |  | 0.25 |  |  | 7.35 |  |  | 1.02 |  |

*Need for Cognition*

|  | **Model 1** | | | **Model 2** | | | **Model 3** | | |
| --- | --- | --- | --- | --- | --- | --- | --- | --- | --- |
|  | *B* | *SEB* | *p* | *B* | *SEB* | *p* | *B* | *SEB* | *p* |
| Intercept | 1.50 | .06 | <.001 | 1.50 | .06 | <.001 | 1.52 | .06 | <.001 |
| Attitude | -.03 | .06 | .614 | -.03 | .06 | .668 | -.03 | .06 | .683 |
| NFC |  |  |  | -.03 | .06 | .653 | -.03 | .06 | .603 |
| Attitude×NFC |  |  |  |  |  |  | -.12 | .06 | .043 |
| R² |  | <.01 |  |  | <.01 |  |  | .01 |  |
| Adjusted R² |  | <.01 |  |  | <.01 |  |  | <.01 |  |
| *F* for change in R² |  | 0.25 |  |  | 0.20 |  |  | 4.11 |  |

*Dark Factor*

|  | **Model 1** | | | **Model 2** | | | **Model 3** | | |
| --- | --- | --- | --- | --- | --- | --- | --- | --- | --- |
|  | *B* | *SEB* | *p* | *B* | *SEB* | *p* | *B* | *SEB* | *p* |
| Intercept | 1.50 | .06 | <.001 | 1.50 | .06 | <.001 | 1.51 | .06 | <.001 |
| Attitude | -.03 | .06 | .614 | -.03 | .06 | .590 | -.03 | .06 | .589 |
| Dark Factor |  |  |  | -.02 | .06 | .775 | -.02 | .06 | .780 |
| Attitude×Dark Factor |  |  |  |  |  |  | .08 | .06 | .212 |
| R² |  | <.01 |  |  | <.01 |  |  | <.01 |  |
| Adjusted R² |  | <.01 |  |  | <.01 |  |  | <.01 |  |
| *F* for change in R² |  | 0.25 |  |  | 0.08 |  |  | 1.56 |  |

**Tables for additional regression analyses including the other variables of interest as covariates in the first step**

**a. Directional Bias**

*Numeracy*

|  | **Model 1** | | | **Model 2** | | | **Model 3** | | | **Model 4** | | |
| --- | --- | --- | --- | --- | --- | --- | --- | --- | --- | --- | --- | --- |
|  | *B* | *SEB* | *p* | *B* | *SEB* | *p* | *B* | *SEB* | *p* | *B* | *SEB* | *p* |
| Intercept | -.24 | .09 | .006 | -.24 | .09 | .006 | -.24 | .09 | .006 | -.26 | .09 | .004 |
| NFC | -.03 | .09 | .758 | -.05 | .09 | .550 | -.01 | .09 | .924 | -.01 | .09 | .937 |
| Dark Factor | -.10 | .09 | .246 | -.08 | .09 | .375 | -.07 | .09 | .412 | -.06 | .09 | .506 |
| Attitude |  |  |  | .18 | .09 | .044 | .20 | .09 | .029 | .21 | .09 | .019 |
| Numeracy |  |  |  |  |  |  | -.21 | .09 | .023 | .20 | .09 | .023 |
| Attitude×Numeracy |  |  |  |  |  |  |  |  |  | .15 | .08 | .084 |
| R² |  | <.01 |  |  | .01 |  |  | .02 |  |  | .02 |  |
| Adjusted R² |  | <.01 |  |  | <.01 |  |  | .01 |  |  | .02 |  |
| *F* for change in R² |  | 0.70 |  |  | 4.06 |  |  | 5.23 |  |  | 2.99 |  |

*Need for Cognition*

|  | **Model 1** | | | **Model 2** | | | **Model 3** | | | **Model 4** | | |
| --- | --- | --- | --- | --- | --- | --- | --- | --- | --- | --- | --- | --- |
|  | *B* | *SEB* | *p* | *B* | *SEB* | *p* | *B* | *SEB* | *p* | *B* | *SEB* | *p* |
| Intercept | -.24 | .09 | .006 | -.24 | .09 | .006 | -.24 | .09 | .006 | -.25 | .09 | .005 |
| Numeracy | -.19 | .09 | .034 | -.21 | .09 | .018 | -.21 | .09 | .023 | -.21 | .09 | .023 |
| Dark Factor | -.10 | .09 | .254 | -.07 | .09 | .414 | -.07 | .09 | .412 | -.07 | .09 | .417 |
| Attitude |  |  |  | .20 | .09 | .028 | .20 | .09 | .029 | .20 | .09 | .029 |
| NFC |  |  |  |  |  |  | -.01 | .09 | .924 | -.01 | .09 | .936 |
| Attitude×NFC |  |  |  |  |  |  |  |  |  | .04 | .08 | .642 |
| R² |  | .01 |  |  | .02 |  |  | .02 |  |  | .02 |  |
| Adjusted R² |  | .01 |  |  | .01 |  |  | .01 |  |  | .01 |  |
| *F* for change in R² |  | 2.92 |  |  | 4.85 |  |  | 0.01 |  |  | 0.22 |  |

*Dark Factor*

|  | **Model 1** | | | **Model 2** | | | **Model 3** | | | **Model 4** | | |
| --- | --- | --- | --- | --- | --- | --- | --- | --- | --- | --- | --- | --- |
|  | *B* | *SEB* | *p* | *B* | *SEB* | *p* | *B* | *SEB* | *p* | *B* | *SEB* | *p* |
| Intercept | -.24 | .09 | .006 | -.24 | .09 | .006 | -.24 | .09 | .006 | -.24 | .09 | .007 |
| Numeracy | -.19 | .09 | .033 | -.21 | .09 | .021 | -.21 | .09 | .023 | -.21 | .09 | .024 |
| NFC | .03 | .09 | .785 | <.01 | .09 | .967 | -.01 | .09 | .924 | -.01 | .09 | .924 |
| Attitude |  |  |  | .21 | .09 | .020 | .20 | .09 | .029 | .20 | .09 | .029 |
| Dark Factor |  |  |  |  |  |  | -.07 | .09 | .412 | -.07 | .09 | .412 |
| Attitude×Dark Factor |  |  |  |  |  |  |  |  |  | .01 | .09 | .918 |
| R² |  | .01 |  |  | .02 |  |  | .02 |  |  | .02 |  |
| Adjusted R² |  | .01 |  |  | .01 |  |  | .01 |  |  | .01 |  |
| *F* for change in R² |  | 2.30 |  |  | 5.42 |  |  | 0.68 |  |  | 0.01 |  |

**b. Absolute Bias**

*Numeracy*

|  | **Model 1** | | | **Model 2** | | | **Model 3** | | | **Model 4** | | |
| --- | --- | --- | --- | --- | --- | --- | --- | --- | --- | --- | --- | --- |
|  | *B* | *SEB* | *p* | *B* | *SEB* | *p* | *B* | *SEB* | *p* | *B* | *SEB* | *p* |
| Intercept | 1.50 | .06 | <.001 | 1.50 | .06 | <.001 | 1.50 | .06 | <.001 | 1.51 | .06 | <.001 |
| NFC | -.03 | .06 | .589 | -.03 | .06 | .641 | .01 | .06 | .910 | .01 | .06 | .918 |
| Dark Factor | -.02 | .06 | .800 | -.02 | .06 | .754 | -.02 | .06 | .815 | -.02 | .06 | .745 |
| Attitude |  |  |  | -.03 | .06 | .642 | -.02 | .06 | .797 | -.02 | .06 | .722 |
| Numeracy |  |  |  |  |  |  | -.17 | .06 | .008 | -.17 | .06 | .008 |
| Attitude×Numeracy |  |  |  |  |  |  |  |  |  | -.06 | .06 | .302 |
| R² |  | <.01 |  |  | <.01 |  |  | .01 |  |  | .02 |  |
| Adjusted R² |  | <.01 |  |  | <.01 |  |  | .01 |  |  | .01 |  |
| *F* for change in R² |  | 0.17 |  |  | 0.22 |  |  | 7.09 |  |  | 1.07 |  |

*Need for Cognition*

|  | **Model 1** | | | **Model 2** | | | **Model 3** | | | **Model 4** | | |
| --- | --- | --- | --- | --- | --- | --- | --- | --- | --- | --- | --- | --- |
|  | *B* | *SEB* | *p* | *B* | *SEB* | *p* | *B* | *SEB* | *p* | *B* | *SEB* | *p* |
| Intercept | 1.50 | .06 | <.001 | 1.50 | .06 | <.001 | 1.50 | .06 | <.001 | 1.52 | .06 | <.001 |
| Numeracy | -.17 | .06 | .006 | -.17 | .06 | .007 | -.17 | .06 | .008 | -.17 | .06 | .007 |
| Dark Factor | -.01 | .06 | .834 | -.02 | .06 | .808 | -.02 | .06 | .815 | -.02 | .06 | .788 |
| Attitude |  |  |  | -.02 | .06 | .806 | -.02 | .06 | .797 | -.02 | .06 | .811 |
| NFC |  |  |  |  |  |  | .01 | .06 | .910 | <.01 | .06 | .962 |
| Attitude×NFC |  |  |  |  |  |  |  |  |  | -.12 | .06 | .038 |
| R² |  | .01 |  |  | .01 |  |  | .01 |  |  | .02 |  |
| Adjusted R² |  | .01 |  |  | .01 |  |  | .01 |  |  | .01 |  |
| *F* for change in R² |  | 3.80 |  |  | 0.60 |  |  | 0.01 |  |  | 4.32 |  |

*Dark Factor*

|  | **Model 1** | | | **Model 2** | | | **Model 3** | | | **Model 4** | | |
| --- | --- | --- | --- | --- | --- | --- | --- | --- | --- | --- | --- | --- |
|  | *B* | *SEB* | *p* | *B* | *SEB* | *p* | *B* | *SEB* | *p* | *B* | *SEB* | *p* |
| Intercept | 1.50 | .06 | <.001 | 1.50 | .06 | <.001 | 1.50 | .06 | <.001 | 1.51 | .06 | <.001 |
| Numeracy | -.17 | .06 | .007 | -.17 | .06 | .008 | -.17 | .06 | .008 | -.16 | .06 | .010 |
| NFC | .01 | .06 | .921 | .01 | .06 | .898 | .01 | .06 | .910 | .01 | .06 | .910 |
| Attitude |  |  |  | -.01 | .06 | .820 | -.02 | .06 | .797 | -.02 | .06 | .790 |
| Dark Factor |  |  |  |  |  |  | -.02 | .06 | .815 | -.01 | .06 | .818 |
| Attitude×Dark Factor |  |  |  |  |  |  |  |  |  | .06 | .06 | .294 |
| R² |  | .01 |  |  | .01 |  |  | .01 |  |  | .02 |  |
| Adjusted R² |  | .01 |  |  | .01 |  |  | .01 |  |  | .01 |  |
| *F* for change in R² |  | 3.78 |  |  | 0.05 |  |  | 0.06 |  |  | 1.10 |  |

**Tables for additional regression analyses checking for higher-order interactions involving attitude**

**a. Directional Bias**

|  | **Model 1** | | | **Model 2** | | | **Model 3** | | | **Model 4** | | |
| --- | --- | --- | --- | --- | --- | --- | --- | --- | --- | --- | --- | --- |
|  | *B* | *SEB* | *p* | *B* | *SEB* | *p* | *B* | *SEB* | *p* | *B* | *SEB* | *p* |
| Intercept | -.24 | .09 | .006 | -.26 | .09 | .004 | -.25 | .09 | .007 | -.26 | .09 | .006 |
| Attitude | .20 | .09 | .029 | .21 | .09 | .019 | .21 | .09 | .021 | .23 | .09 | .014 |
| Numeracy | -.21 | .09 | .023 | -.20 | .09 | .024 | -.21 | .09 | .024 | -.21 | .09 | .023 |
| NFC | -.01 | .09 | .924 | -.01 | .09 | .937 | -.02 | .09 | .815 | -.01 | .09 | .906 |
| Dark Factor | -.07 | .09 | .412 | -.06 | .09 | .507 | -.07 | .09 | .458 | -.06 | .09 | .496 |
| Att.×Num |  |  |  | .15 | .09 | .098 | .15 | .09 | .085 | .14 | .09 | .113 |
| Att.×NFC |  |  |  | .001 | .09 | .991 | -.01 | .09 | .928 | -.01 | .09 | .946 |
| Att.×DF |  |  |  | .003 | .09 | .968 | .02 | .09 | .815 | .01 | .09 | .889 |
| DF×Num |  |  |  |  |  |  | -.02 | .09 | .863 | -.03 | .09 | .785 |
| NFC×Num |  |  |  |  |  |  | -.06 | .09 | .483 | -.08 | .09 | .411 |
| DF×NFC |  |  |  |  |  |  | -.11 | .09 | .222 | -.11 | .09 | .216 |
| Att.×Num×DF |  |  |  |  |  |  |  |  |  | -.10 | .09 | .249 |
| Att.×NFC×DF |  |  |  |  |  |  |  |  |  | .01 | .08 | .896 |
| Att.×NFC×Num |  |  |  |  |  |  |  |  |  | -.07 | .08 | .406 |
| R² |  | .02 |  |  | .02 |  |  | .03 |  |  | .03 |  |
| Adjusted R² |  | .01 |  |  | .01 |  |  | .01 |  |  | .01 |  |
| *F* for change in R² |  | 2.68 |  |  | 1.00 |  |  | 0.74 |  |  | 0.62 |  |

**b. Absolute Bias**

|  | **Model 1** | | | **Model 2** | | | **Model 3** | | | **Model 4** | | |
| --- | --- | --- | --- | --- | --- | --- | --- | --- | --- | --- | --- | --- |
|  | *B* | *SEB* | *p* | *B* | *SEB* | *p* | *B* | *SEB* | *p* | *B* | *SEB* | *p* |
| Intercept | 1.50 | .06 | <.001 | 1.53 | .06 | <.001 | 1.55 | .06 | <.001 | 1.55 | .06 | <.001 |
| Attitude | -.02 | .06 | .797 | -.02 | .06 | .762 | -.03 | .06 | .696 | -.02 | .07 | .760 |
| Numeracy | -.17 | .06 | .008 | -.17 | .06 | .009 | -.18 | .06 | .006 | -.17 | .07 | .009 |
| NFC | .01 | .06 | .910 | <.01 | .06 | .960 | .01 | .06 | .859 | .01 | .07 | .893 |
| Dark Factor | -.02 | .06 | .815 | -.02 | .06 | .755 | -.02 | .06 | .795 | -.01 | .06 | .864 |
| Att.×Num |  |  |  | -.03 | .06 | .576 | -.03 | .06 | .625 | -.03 | .06 | .612 |
| Att.×NFC |  |  |  | -.11 | .06 | .080 | -.09 | .06 | .170 | -.09 | .06 | .151 |
| Att.×DF |  |  |  | .05 | .06 | .379 | .05 | .06 | .423 | .05 | .06 | .435 |
| DF×Num |  |  |  |  |  |  | -.07 | .06 | .304 | -.07 | .07 | .308 |
| NFC×Num |  |  |  |  |  |  | -.10 | .06 | .133 | -.11 | .07 | .108 |
| DF×NFC |  |  |  |  |  |  | .06 | .06 | .303 | .06 | .06 | .312 |
| Att.×Num×DF |  |  |  |  |  |  |  |  |  | .01 | .06 | .877 |
| Att.×NFC×DF |  |  |  |  |  |  |  |  |  | -.04 | .06 | .546 |
| Att.×NFC×Num |  |  |  |  |  |  |  |  |  | -.03 | .06 | .604 |
| R² |  | .01 |  |  | .02 |  |  | .03 |  |  | .03 |  |
| Adjusted R² |  | .01 |  |  | .01 |  |  | .01 |  |  | .01 |  |
| *F* for change in R² |  | 1.91 |  |  | 1.78 |  |  | 1.22 |  |  | 0.21 |  |

**S10 Predictors of study evaluation accuracy**

In order to test whether the four factors under investigation (numeracy, Need for Cognition, Dark Factor of Personality, attitude strength) influenced the likelihood that the study results were interpreted incorrectly (0) or correctly (1), we ran logistic regression analyses. These logistic regression analyses contained attitude towards stricter regulations to reduce CO_2_ emissions and one of the four factors under investigation (and their interaction) as predictors (z-standardized). We ran two logistic regression analyses for each factor under investigation, one including pro-regulation study evaluation accuracy and one including anti-regulation study evaluation accuracy as the criterion.

**Numeracy**

|  | Pro-Regulation Study | | | Anti-Regulation Study | | |
| --- | --- | --- | --- | --- | --- | --- |
|  | OR | CI | p | OR | CI | p |
| Attitude | 1.09 | 0.92, 1.29 | .340 | 0.86 | 0.72, 1.02 | .086 |
| Numeracy | 1.31 | 1.10, 1.55 | .002 | 1.65 | 1.37, 1.97 | <.001 |
| Interaction | 1.09 | 0.92, 1.28 | .332 | 0.88 | 0.74, 1.05 | .151 |

**Need for Cognition**

|  | Pro-Regulation Study | | | Anti-Regulation Study | | |
| --- | --- | --- | --- | --- | --- | --- |
|  | OR | CI | p | OR | CI | p |
| Attitude | 1.06 | 0.89, 1.25 | .526 | 0.88 | 0.74, 1.05 | .152 |
| NFC | 1.37 | 1.15, 1.64 | <.001 | 1.36 | 1.14, 1.62 | .001 |
| Interaction | 1.07 | 0.91, 1.26 | .390 | 1.04 | 0.89, 1.23 | .621 |

**Dark Factor of Personality**

|  | Pro-Regulation Study | | | Anti-Regulation Study | | |
| --- | --- | --- | --- | --- | --- | --- |
|  | OR | CI | p | OR | CI | p |
| Attitude | 1.09 | 0.92, 1.29 | .337 | 0.92 | 0.78, 1.09 | .342 |
| Dark Factor | 0.87 | 0.73, 1.03 | .095 | 0.98 | 0.82, 1.16 | .792 |
| Interaction | 0.92 | 0.78, 1.09 | .322 | 0.89 | 0.75, 1.05 | .156 |

**Attitude strength**

|  | Pro-Regulation Study | | | Anti-Regulation Study | | |
| --- | --- | --- | --- | --- | --- | --- |
|  | OR | CI | p | OR | CI | p |
| Attitude | 0.95 | 0.77, 1.18 | .655 | 0.89 | 0.71, 1.11 | .286 |
| Attitude strength | 1.26 | 1.01, 1.56 | .041 | 1.07 | 0.86, 1.33 | .527 |
| Interaction | 1.19 | 0.99, 1.42 | .064 | 0.94 | 0.79, 1.13 | .516 |

**S11 Additional information about the stimulus material**

As outlined in the manuscript, the numbers in the contingency tables were designed in a way so that superficial processing (i.e., comparing absolute numbers between two cells instead of ratios) easily leads to the wrong interpretation. In the manuscript, this was described for the pro-regulation study in Stimulus Set A. However, the logic behind the numbers in the anti-regulation study is exactly the same, leading to the opposite conclusion: Here, there are fewer cities/districts with stricter regulations to reduce CO_2_ emissions (97) than cities/districts without stricter regulations to reduce CO_2_ emissions (205) that show an increase in CO_2_ emissions, which can lead to the wrong conclusion that stricter regulations are effective. In fact, however, there is an increase in CO_2_ emissions in 97 out of 116 cities/districts with stricter regulations (83.6%) compared to an increase in CO_2_ emissions in 205 out of 274 cities/districts without stricter regulations (74.8%), indicating that stricter regulations are an overall counterproductive intervention.

**STUDY 2**

**S12 Attention check items**

Session 1 included two attention check items, one intermixed with the items on the scale measuring NFC (“It’s important that you pay attention to this study. Please tick ‘very strong disagreement’”) and one intermixed with the items on the scale measuring D (“It’s important that you pay attention to this study. Please tick ‘strongly agree’”). In session 2, participants also responded to two attention check items. One attention check item (“It’s important that you pay attention to this study. Please tick ‘extremely important’”) was intermixed with the items regarding the attitude towards stricter regulations to reduce CO_2_ emissions. The second attention check item was presented after evaluating the two fictitious studies in order to ensure that the participants had read the instructions carefully (“The two studies on the effects of stricter regulations to reduce CO_2_ emissions were conducted: (a) in two states of the US: The names of the states were not discloses; (b) in two states of the US: Ohio and Michigan; (c) in two European countries: France and Germany; (d) in two European countries: The names of the countries were not disclosed”).

**S13 Selection of participants for the Main Study (Session 2)**

From the 1604 participants who completed Session 1, 1450 participants were invited for participation in Session 2. We decided not to invite participants for Session 2 who had failed one of the attention check items (*N* = 97) or who had noticeable short (i.e., less than one third of the median response time; *N* = 1) or long (i.e., more than six times the median response time; *N* = 1) response times indicating careless responding. We also decided to exclude two more participants from further participation, as they had indicated an unreasonably low (4) or high (774) age. The subset of 1450 participants was drawn from the remaining participants following the procedure described in the main text.

**S14 Distribution of the participants with respect to their attitude towards stricter regulations to reduce CO_2_ emissions in the final sample (*****N* = 1198)**

| **Attitude towards stricter regulations to reduce CO_2_ emissions** | **Number of participants** |
| --- | --- |
| 1 = extremely oppose | 88 |
| 2 | 102 |
| 3 | 171 |
| 4 | 166 |
| 5 | 360 |
| 6 | 233 |
| 7 = extremely favor | 78 |

**S15 Ethnicity**

| **Ethnicity** | **Number of participants** |
| --- | --- |
| White | 1066 |
| Black/Black British | 29 |
| Asian/Asian British | 63 |
| Mixed | 28 |
| Other | 9 |
| Prefer not to say | 3 |

**S16 Education**

| **Education** | **Number of participants** |
| --- | --- |
| Primary school | 0 |
| Secondary school up to 16 years | 104 |
| Higher or secondary or further education (A-levels, BTEC, etc.) | 224 |
| College or university | 585 |
| Post-graduate degree | 281 |
| Other | 3 |
| Prefer not to say | 1 |

**S17 Political Orientation**

| **Political orientation** | **Number of participants** |
| --- | --- |
| Extremely left | 53 |
| Left | 323 |
| Center left | 279 |
| Center | 322 |
| Center right | 148 |
| Right | 59 |
| Extremely right | 14 |

**S18 Analysis of potential order effects**

*Means and Standard Deviations (in parentheses) of Directional Bias and Absolute Bias as a Function of Study Order (Pro-Regulation Study first, Anti-Regulation Study second vs. Anti-Regulation Study first, Pro-Regulation Study second) and Study Number Mapping (Pro-Regulation Study with N_cities/districts_ = 426, Anti-Regulation Study with N_cities/districts_ = 390 vs. Pro-Regulation Study with N_cities/districts_ = 390, Anti-Regulation Study with N_cities/districts_ = 426)*

|  | *N* | Directional Bias | Absolute Bias |
| --- | --- | --- | --- |
| Study Order: Pro-Regulation Study first, Anti-Regulation Study second |  |  |  |
| Study Number Mapping: |  |  |  |
| Pro-Regulation Study with *N* = 426,  Anti-Regulation Study with *N* = 390 | 295 | -0.38 (2.08) | 1.50 (1.50) |
| Pro-Regulation Study with *N* = 390,  Anti-Regulation Study with *N* = 426 | 300 | -0.60 (2.08) | 1.58 (1.48) |
| Study Order: Anti-Regulation Study first, Pro-Regulation Study second |  |  |  |
| Study Number Mapping: |  |  |  |
| Pro-Regulation Study with *N* = 426,  Anti-Regulation Study with *N* = 390 | 299 | -0.30 (2.09) | 1.52 (1.46) |
| Pro-Regulation Study with *N* = 390,  Anti-Regulation Study with *N* = 426 | 304 | -0.54 (2.13) | 1.65 (1.44) |

*Statistics from the univariate 2 (Study Order: Pro-Regulation Study first, Anti-Regulation Study second vs. Anti-Regulation Study first, Pro-Regulation Study second) x 2 (Study Number Mapping: Pro-Regulation Study with N_cities/districts_ = 426, Anti-Regulation Study with N_cities/districts_ = 390 vs. Pro-Regulation Study with N_cities/districts_ = 390, Anti-Regulation Study with N_cities/districts_ = 426) ANOVAs on Directional Bias and Absolute Bias.*

|  | Directional Bias | | | Absolute Bias | | |
| --- | --- | --- | --- | --- | --- | --- |
|  | *F*  (1, 413) | *P* | η_p_^2^ | *F*  (1, 413) | *p* | η_p_^2^ |
| Main Effect Study Order | 0.33 | .565 | <.001 | 0.38 | .537 | <.001 |
| Main Effect Study Number Mapping | 0.02 | .897 | <.001 | 0.09 | .763 | <.001 |
| Interaction Study Order * Study Number Mapping | 3.59 | .058 | .003 | 1.60 | .206 | .001 |

**S19 Results for attitude strength**

To investigate our research questions, we ran separate hierarchical regression analyses for each factor potentially influencing motivated reasoning. The predictors in each regression analysis were the attitude towards stricter regulations to reduce CO2 emissions (z-standardized, Step 1), the respective factor potentially influencing motivated reasoning (z-standardized, entered in Step 2), and the product term of the two variables (entered in Step 3). We ran separate regression analyses for the directional bias and the absolute bias as the criterion.

***Directional Bias***

Attitude strength as the second predictor in the equation yielded no significant effect, with *B* = -.13, *SEB* = .07, *p* = .006, Δ*R^2^* = .003, for the association, and *B* = .05, *SEB* = .09, *p* = .413, Δ*R^2^* = .001, for the interaction.

***Absolute Bias***

Likewise, when absolute bias served as the criterion, attitude strength as the second predictor in the equation yielded no significant effect, with *B* = .05, *SEB* = .05, *p* = .276, Δ*R^2^* = .001, for the association, and *B* = .01, *SEB* = .04, *p* = .883, Δ*R^2^* < .001 for the interaction.

**S20 Additional tables for regression analyses**

**Tables for regression analyses reported in the manuscript**

**a. Directional Bias**

*Numeracy*

|  | **Model 1** | | | **Model 2** | | | **Model 3** | | |
| --- | --- | --- | --- | --- | --- | --- | --- | --- | --- |
|  | *B* | *SEB* | *p* | *B* | *SEB* | *p* | *B* | *SEB* | *p* |
| Intercept | -.46 | .06 | <.001 | -.46 | .06 | <.001 | -.46 | .06 | <.001 |
| Attitude | .23 | .06 | <.001 | .23 | .06 | <.001 | .23 | .06 | <.001 |
| Numeracy |  |  |  | -.06 | .06 | .311 | -.06 | .06 | .315 |
| Attitude×Numeracy |  |  |  |  |  |  | .01 | .06 | .907 |
| R² |  | .01 |  |  | .01 |  |  | .01 |  |
| Adjusted R² |  | .01 |  |  | .01 |  |  | .01 |  |
| *F* for change in R² |  | 14.29 |  |  | 1.03 |  |  | 0.01 |  |

*Need for Cognition*

|  | **Model 1** | | | **Model 2** | | | **Model 3** | | |
| --- | --- | --- | --- | --- | --- | --- | --- | --- | --- |
|  | *B* | *SEB* | *p* | *B* | *SEB* | *p* | *B* | *SEB* | *p* |
| Intercept | -.46 | .06 | <.001 | -.46 | .06 | <.001 | -.45 | .06 | <.001 |
| Attitude | .23 | .06 | <.001 | .23 | .06 | <.001 | .23 | .06 | <.001 |
| NFC |  |  |  | .01 | .06 | .908 | .01 | .06 | .921 |
| Attitude×NFC |  |  |  |  |  |  | -.01 | .06 | .832 |
| R² |  | .01 |  |  | .01 |  |  | .01 |  |
| Adjusted R² |  | .01 |  |  | .01 |  |  | .01 |  |
| *F* for change in R² |  | 14.29 |  |  | 0.01 |  |  | 0.05 |  |

*Dark Factor*

|  | **Model 1** | | | **Model 2** | | | **Model 3** | | |
| --- | --- | --- | --- | --- | --- | --- | --- | --- | --- |
|  | *B* | *SEB* | *p* | *B* | *SEB* | *p* | *B* | *SEB* | *p* |
| Intercept | -.46 | .06 | <.001 | -.46 | .06 | <.001 | -.45 | .06 | <.001 |
| Attitude | .23 | .06 | <.001 | .24 | .06 | <.001 | .23 | .06 | <.001 |
| Dark Factor |  |  |  | .07 | .06 | .233 | .07 | .06 | .252 |
| Attitude×Dark Factor |  |  |  |  |  |  | .06 | .06 | .275 |
| R² |  | .01 |  |  | .01 |  |  | .01 |  |
| Adjusted R² |  | .01 |  |  | .01 |  |  | .01 |  |
| *F* for change in R² |  | 14.29 |  |  | 1.42 |  |  | 1.20 |  |

**b. Absolute Bias**

*Numeracy*

|  | **Model 1** | | | **Model 2** | | | **Model 3** | | |
| --- | --- | --- | --- | --- | --- | --- | --- | --- | --- |
|  | *B* | *SEB* | *p* | *B* | *SEB* | *p* | *B* | *SEB* | *p* |
| Intercept | 1.56 | .04 | <.001 | 1.56 | .04 | <.001 | 1.57 | .06 | <.001 |
| Attitude | -.11 | .04 | .011 | -.09 | .04 | .029 | -.10 | .06 | .019 |
| Numeracy |  |  |  | -.18 | .04 | <.001 | -.19 | .06 | <.001 |
| Attitude×Numeracy |  |  |  |  |  |  | -.07 | .06 | .091 |
| R² |  | .01 |  |  | .02 |  |  | .02 |  |
| Adjusted R² |  | .01 |  |  | .02 |  |  | .02 |  |
| *F* for change in R² |  | 6.44 |  |  | 18.62 |  |  | 2.87 |  |

*Need for Cognition*

|  | **Model 1** | | | **Model 2** | | | **Model 3** | | |
| --- | --- | --- | --- | --- | --- | --- | --- | --- | --- |
|  | *B* | *SEB* | *p* | *B* | *SEB* | *p* | *B* | *SEB* | *p* |
| Intercept | 1.56 | .04 | <.001 | 1.55 | .04 | <.001 | 1.57 | .04 | <.001 |
| Attitude | -.11 | .04 | .011 | -.10 | .04 | .016 | -.10 | .04 | .018 |
| NFC |  |  |  | -.03 | .04 | .461 | -.04 | .04 | .419 |
| Attitude×NFC |  |  |  |  |  |  | -.04 | .04 | .337 |
| R² |  | .01 |  |  | .01 |  |  | .01 |  |
| Adjusted R² |  | .01 |  |  | <.01 |  |  | <.01 |  |
| *F* for change in R² |  | 6.44 |  |  | 0.54 |  |  | 0.92 |  |

*Dark Factor*

|  | **Model 1** | | | **Model 2** | | | **Model 3** | | |
| --- | --- | --- | --- | --- | --- | --- | --- | --- | --- |
|  | *B* | *SEB* | *p* | *B* | *SEB* | *p* | *B* | *SEB* | *p* |
| Intercept | 1.56 | .04 | <.001 | 1.56 | .04 | <.001 | 1.56 | .04 | <.001 |
| Attitude | -.11 | .04 | .011 | -.11 | .04 | .007 | -.11 | .04 | .007 |
| Dark Factor |  |  |  | -.06 | .04 | .144 | -.06 | .04 | .144 |
| Attitude×Dark Factor |  |  |  |  |  |  | .001 | .04 | .986 |
| R² |  | .01 |  |  | .01 |  |  | .01 |  |
| Adjusted R² |  | .01 |  |  | .01 |  |  | .01 |  |
| *F* for change in R² |  | 6.44 |  |  | 2.12 |  |  | <0.01 |  |

**Tables for additional regression analyses including the other variables of interest as covariates in the first step**

**a. Directional Bias**

*Numeracy*

|  | **Model 1** | | | **Model 2** | | | **Model 3** | | | **Model 4** | | |
| --- | --- | --- | --- | --- | --- | --- | --- | --- | --- | --- | --- | --- |
|  | *B* | *SEB* | *p* | *B* | *SEB* | *p* | *B* | *SEB* | *p* | *B* | *SEB* | *p* |
| Intercept | -.46 | .06 | <.001 | -.46 | .06 | <.001 | -.46 | .06 | <.001 | -.46 | .06 | <.001 |
| NFC | .05 | .06 | .459 | .02 | .06 | .792 | .03 | .06 | .604 | .03 | .06 | .599 |
| Dark Factor | .05 | .06 | .392 | .07 | .06 | .224 | .08 | .06 | .191 | .08 | .06 | .186 |
| Attitude |  |  |  | .23 | .06 | <.001 | .24 | .06 | <.001 | .24 | .06 | <.001 |
| Numeracy |  |  |  |  |  |  | -.07 | .06 | .243 | -.07 | .06 | .248 |
| Attitude×Numeracy |  |  |  |  |  |  |  |  |  | .01 | .06 | .817 |
| R² |  | <.01 |  |  | .01 |  |  | .01 |  |  | .01 |  |
| Adjusted R² |  | <.01 |  |  | .01 |  |  | .01 |  |  | .01 |  |
| *F* for change in R² |  | 0.57 |  |  | 14.63 |  |  | 1.36 |  |  | 0.05 |  |

*Need for Cognition*

|  | **Model 1** | | | **Model 2** | | | **Model 3** | | | **Model 4** | | |
| --- | --- | --- | --- | --- | --- | --- | --- | --- | --- | --- | --- | --- |
|  | *B* | *SEB* | *p* | *B* | *SEB* | *p* | *B* | *SEB* | *p* | *B* | *SEB* | *p* |
| Intercept | -.46 | .06 | <.001 | -.46 | .06 | <.001 | -.46 | .06 | <.001 | -.45 | .06 | <.001 |
| Numeracy | -.04 | .06 | .471 | -.07 | .06 | .281 | -.07 | .06 | .243 | -.07 | .06 | .239 |
| Dark Factor | .05 | .06 | .427 | .08 | .06 | .212 | .08 | .06 | .191 | .08 | .06 | .187 |
| Attitude |  |  |  | .24 | .06 | <.001 | .24 | .06 | <.001 | .239 | .06 | <.001 |
| NFC |  |  |  |  |  |  | .03 | .06 | .604 | .03 | .06 | .618 |
| Attitude×NFC |  |  |  |  |  |  |  |  |  | -.02 | .06 | .758 |
| R² |  | <.01 |  |  | .01 |  |  | .01 |  |  | .01 |  |
| Adjusted R² |  | <.01 |  |  | .01 |  |  | .01 |  |  | .01 |  |
| *F* for change in R² |  | 0.55 |  |  | 15.77 |  |  | 0.27 |  |  | 0.10 |  |

*Dark Factor*

|  | **Model 1** | | | **Model 2** | | | **Model 3** | | | **Model 4** | | |
| --- | --- | --- | --- | --- | --- | --- | --- | --- | --- | --- | --- | --- |
|  | *B* | *SEB* | *p* | *B* | *SEB* | *p* | *B* | *SEB* | *p* | *B* | *SEB* | *p* |
| Intercept | -.46 | .06 | <.001 | -.46 | .06 | <.001 | -.46 | .06 | <.001 | -.45 | .06 | <.001 |
| Numeracy | -.05 | .06 | .395 | -.07 | .06 | .288 | -.07 | .06 | .243 | -.07 | .06 | .279 |
| NFC | .05 | .06 | .421 | .02 | .06 | .732 | .03 | .06 | .604 | .03 | .06 | .651 |
| Attitude |  |  |  | .23 | .06 | <.001 | .24 | .06 | <.001 | .24 | .06 | <.001 |
| Dark Factor |  |  |  |  |  |  |  | .06 | .191 | .08 | .06 | .211 |
| Attitude×Dark Factor |  |  |  |  |  |  |  |  |  | .05 | .06 | .323 |
| R² |  | <.01 |  |  | .01 |  |  | .01 |  |  | .02 |  |
| Adjusted R² |  | <.01 |  |  | .01 |  |  | .01 |  |  | .01 |  |
| *F* for change in R² |  |  |  |  |  |  |  |  |  |  |  |  |

**b. Absolute Bias**

*Numeracy*

|  | **Model 1** | | | **Model 2** | | | **Model 3** | | | **Model 4** | | |
| --- | --- | --- | --- | --- | --- | --- | --- | --- | --- | --- | --- | --- |
|  | *B* | *SEB* | *p* | *B* | *SEB* | *p* | *B* | *SEB* | *p* | *B* | *SEB* | *p* |
| Intercept | 1.56 | .04 | <.001 | 1.56 | .04 | <.001 | 1.56 | .04 | <.001 | 1.57 | .04 | <.001 |
| NFC | -.05 | .04 | .212 | -.04 | .04 | .356 | <.01 | .04 | .981 | <.01 | .04 | .968 |
| Dark Factor | -.06 | .04 | .184 | -.07 | .04 | .118 | -.05 | .04 | .219 | -.06 | .04 | .171 |
| Attitude |  |  |  | -.11 | .04 | .011 | -.10 | .04 | .022 | -.11 | .04 | .013 |
| Numeracy |  |  |  |  |  |  | -.18 | .04 | <.001 | -.18 | .04 | <.001 |
| Attitude×Numeracy |  |  |  |  |  |  |  |  |  | -.07 | .04 | .073 |
| R² |  | <.01 |  |  | .01 |  |  | .02 |  |  | .03 |  |
| Adjusted R² |  | <.01 |  |  | .01 |  |  | .02 |  |  | .02 |  |
| *F* for change in R² |  | 1.46 |  |  | 6.50 |  |  | 17.13 |  |  | 3.22 |  |

*Need for Cognition*

|  | **Model 1** | | | **Model 2** | | | **Model 3** | | | **Model 4** | | |
| --- | --- | --- | --- | --- | --- | --- | --- | --- | --- | --- | --- | --- |
|  | *B* | *SEB* | *p* | *B* | *SEB* | *p* | *B* | *SEB* | *p* | *B* | *SEB* | *p* |
| Intercept | 1.56 | .04 | <.001 | 1.56 | .04 | <.001 | 1.56 | .04 | <.001 | 1.57 | .04 | <.001 |
| Numeracy | -.19 | .04 | <.001 | -.18 | .04 | <.001 | -.18 | .04 | <.001 | -.18 | .04 | <.001 |
| Dark Factor | -.04 | .04 | .326 | -.05 | .04 | .214 | -.05 | .04 | .219 | -.05 | .04 | .238 |
| Attitude |  |  |  | -.10 | .04 | .021 | -.10 | .04 | .022 | -.10 | .04 | .025 |
| NFC |  |  |  |  |  |  | <.01 | .04 | .981 | <.01 | .04 | .968 |
| Attitude×NFC |  |  |  |  |  |  |  |  |  | -.04 | .04 | .288 |
| R² |  | .02 |  |  | .02 |  |  | .02 |  |  | .02 |  |
| Adjusted R² |  | .02 |  |  | .02 |  |  | .02 |  |  | .02 |  |
| *F* for change in R² |  | 10.64 |  |  | 5.35 |  |  | <0.01 |  |  | 1.13 |  |

*Dark Factor*

|  | **Model 1** | | | **Model 2** | | | **Model 3** | | | **Model 4** | | |
| --- | --- | --- | --- | --- | --- | --- | --- | --- | --- | --- | --- | --- |
|  | *B* | *SEB* | *p* | *B* | *SEB* | *p* | *B* | *SEB* | *p* | *B* | *SEB* | *p* |
| Intercept | 1.56 | .04 | <.001 | 1.56 | .04 | <.001 | 1.56 | .04 | <.001 | 1.56 | .04 | <.001 |
| Numeracy | -.19 | .04 | <.001 | -.18 | .04 | <.001 | -.18 | .04 | <.001 | -.18 | .04 | <.001 |
| NFC | <.01 | .04 | .941 | .01 | .04 | .846 | <.01 | .04 | .981 | <.01 | .04 | .967 |
| Attitude |  |  |  | -.09 | .04 | .029 | -.10 | .04 | .022 | -.10 | .04 | .022 |
| Dark Factor |  |  |  |  |  |  | -.05 | .04 | .219 | -.05 | .04 | .226 |
| Attitude×Dark Factor |  |  |  |  |  |  |  |  |  | -.01 | .04 | .786 |
| R² |  | .02 |  |  | .02 |  |  | .02 |  |  | .02 |  |
| Adjusted R² |  | .02 |  |  | .02 |  |  | .02 |  |  | .02 |  |
| *F* for change in R² |  | 10.15 |  |  | 4.80 |  |  | 1.51 |  |  | 0.07 |  |

**Tables for additional regression analyses checking for higher-order interactions involving attitude**

**a. Directional Bias**

|  | **Model 1** | | | **Model 2** | | | **Model 3** | | | **Model 4** | | |
| --- | --- | --- | --- | --- | --- | --- | --- | --- | --- | --- | --- | --- |
|  | *B* | *SEB* | *p* | *B* | *SEB* | *p* | *B* | *SEB* | *p* | *B* | *SEB* | *p* |
| Intercept | -.46 | .06 | <.001 | -.45 | .06 | <.001 | -.48 | .06 | <.001 | -.47 | .06 | <.001 |
| Attitude | .24 | .06 | <.001 | .24 | .06 | <.001 | .24 | .06 | <.001 | .24 | .07 | <.001 |
| Numeracy | -.07 | .06 | .243 | -.07 | .06 | .279 | -.05 | .06 | .395 | -.05 | .06 | .451 |
| NFC | .03 | .06 | .604 | .03 | .06 | .653 | .03 | .06 | .648 | .03 | .06 | .645 |
| Dark Factor | .08 | .06 | .191 | .08 | .06 | .202 | .07 | .06 | .239 | .09 | .06 | .183 |
| Att.×Num |  |  |  | .02 | .06 | .806 | -.01 | .06 | .819 | -.02 | .06 | .770 |
| Att.×NFC |  |  |  | -.01 | .06 | .815 | -.04 | .06 | .505 | -.05 | .06 | .403 |
| Att.×DF |  |  |  | .05 | .06 | .353 | .10 | .06 | .077 | .12 | .06 | .040 |
| DF×Num |  |  |  |  |  |  | -.06 | .06 | .350 | -.06 | .06 | .313 |
| NFC×Num |  |  |  |  |  |  | .12 | .06 | .042 | .11 | .06 | .062 |
| DF×NFC |  |  |  |  |  |  | -.13 | .06 | .029 | -.12 | .06 | .036 |
| Att.×Num×DF |  |  |  |  |  |  |  |  |  | .07 | .05 | .159 |
| Att.×NFC×DF |  |  |  |  |  |  |  |  |  | -.06 | .04 | .176 |
| Att.×NFC×Num |  |  |  |  |  |  |  |  |  | -.05 | .05 | .360 |
| R² |  | .01 |  |  | .02 |  |  | .02 |  |  | .03 |  |
| Adjusted R² |  | .01 |  |  | .01 |  |  | .02 |  |  | .02 |  |
| *F* for change in R² |  | 4.29 |  |  | 0.36 |  |  | 3.75 |  |  | 1.32 |  |

**b. Absolute Bias**

|  | **Model 1** | | | **Model 2** | | | **Model 3** | | | **Model 4** | | |
| --- | --- | --- | --- | --- | --- | --- | --- | --- | --- | --- | --- | --- |
|  | *B* | *SEB* | *p* | *B* | *SEB* | *p* | *B* | *SEB* | *p* | *B* | *SEB* | *p* |
| Intercept | 1.56 | .04 | <.001 | 1.57 | .04 | <.001 | 1.57 | .04 | <.001 | 1.57 | .04 | <.001 |
| Attitude | -.10 | .04 | .022 | -.10 | .04 | .016 | -.10 | .04 | .021 | -.10 | .05 | .021 |
| Numeracy | -.18 | .04 | <.001 | -.19 | .04 | <.001 | -.19 | .04 | <.001 | -.20 | .04 | <.001 |
| NFC | <.01 | .04 | .981 | <.01 | .04 | .957 | <.01 | .04 | .995 | -.01 | .04 | .906 |
| Dark Factor | -.05 | .04 | .219 | -.06 | .04 | .193 | -.05 | .04 | .236 | -.05 | .05 | .302 |
| Att.×Num |  |  |  | -.07 | .04 | .126 | -.06 | .04 | .185 | -.05 | .04 | .220 |
| Att.×NFC |  |  |  | -.03 | .04 | .524 | -.02 | .04 | .643 | -.01 | .04 | .761 |
| Att.×DF |  |  |  | -.01 | .04 | .772 | -.02 | .04 | .632 | -.03 | .04 | .466 |
| DF×Num |  |  |  |  |  |  | .05 | .04 | .216 | .05 | .04 | .258 |
| NFC×Num |  |  |  |  |  |  | -.02 | .04 | .696 | -.01 | .04 | .833 |
| DF×NFC |  |  |  |  |  |  | .01 | .04 | .723 | .01 | .04 | .868 |
| Att.×Num×DF |  |  |  |  |  |  |  |  |  | -.04 | .04 | .270 |
| Att.×NFC×DF |  |  |  |  |  |  |  |  |  | <.01 | .03 | .997 |
| Att.×NFC×Num |  |  |  |  |  |  |  |  |  | .03 | .04 | .485 |
| R² |  | .02 |  |  | .03 |  |  | .03 |  |  | .03 |  |
| Adjusted R² |  | .02 |  |  | .02 |  |  | .02 |  |  | .02 |  |
| *F* for change in R² |  | 6.67 |  |  | 1.22 |  |  | 0.75 |  |  | 0.74 |  |

**S21 Predictors of study evaluation accuracy**

In order to test whether the four factors under investigation (numeracy, Need for Cognition, Dark Factor of Personality, attitude strength) influenced the likelihood that the study results were interpreted incorrectly (0) or correctly (1), we ran logistic regression analyses. These logistic regression analyses contained attitude towards stricter regulations to reduce CO_2_ emissions and one of the four factors under investigation (and their interaction) as predictors (z-standardized). We ran two logistic regression analyses for each factor under investigation, one including pro-regulation study evaluation accuracy and one including anti-regulation study evaluation accuracy as the criterion.

**Numeracy**

|  | Pro-Regulation Study | | | Anti-Regulation Study | | |
| --- | --- | --- | --- | --- | --- | --- |
|  | OR | CI | p | OR | CI | p |
| Attitude | 1.32 | 1.17, 1.48 | <.001 | 1.01 | 0.89, 1.13 | .940 |
| Numeracy | 1.23 | 1.09, 1.38 | .001 | 1.52 | 1.35, 1.72 | <.001 |
| Interaction | 1.02 | 0.91, 1.14 | .766 | 1.01 | 0.90, 1.13 | .908 |

**Need for Cognition**

|  | Pro-Regulation Study | | | Anti-Regulation Study | | |
| --- | --- | --- | --- | --- | --- | --- |
|  | OR | CI | p | OR | CI | p |
| Attitude | 1.24 | 1.10, 1.39 | <.001 | 1.03 | 0.92, 1.16 | .627 |
| NFC | 1.09 | 0.97, 1.23 | .145 | 1.08 | 0.96, 1.21 | .209 |
| Interaction | 0.95 | 0.85, 1.06 | .335 | 1.00 | 0.90, 1.10 | .924 |

**Dark Factor of Personality**

|  | Pro-Regulation Study | | | Anti-Regulation Study | | |
| --- | --- | --- | --- | --- | --- | --- |
|  | OR | CI | p | OR | CI | p |
| Attitude | 1.25 | 1.11, 1.41 | <.001 | 1.04 | 0.93, 1.17 | .477 |
| Dark Factor | 1.04 | 0.92, 1.16 | .548 | 1.03 | 0.91, 1.15 | .667 |
| Interaction | 1.04 | 0.93, 1.15 | .504 | 0.99 | 0.89, 1.10 | .838 |

**Attitude strength**

|  | Pro-Regulation Study | | | Anti-Regulation Study | | |
| --- | --- | --- | --- | --- | --- | --- |
|  | OR | CI | p | OR | CI | p |
| Attitude | 1.38 | 1.20, 1.58 | <.001 | 1.05 | 0.93, 1.20 | .429 |
| Attitude strength | 0.82 | 0.72, 0.94 | .004 | 0.98 | 0.86, 1.12 | .756 |
| Interaction | 0.96 | 0.86, 1.08 | .479 | 0.90 | 0.81, 1.01 | .072 |

**S22** **Trustworthiness of study results and confidence in one’s interpretation**

Trust in the studies’ results did not differ between the pro-regulation study (*M* = 3.52, *SD* = 1.11) and the anti-regulation study results (*M* = 3.50, *SD* = 1.11), *t*(1197) = 0.87, *p* = .384, *d* = 0.03, providing further evidence for the validity of our stimulus material.

Confidence in the interpretation of the pro-regulation study results (*M* = 4.11, *SD* = 1.31) was slightly higher than confidence in the interpretation of the anti-regulation study results (*M* = 4.02, *SD* = 1.31), *t*(1197) = 2.88, *p* = .004, *d* = 0.08.

Furthermore, we examined whether confidence in one’s interpretation was related to degree of bias. On the one hand, one may argue that non-biased responders may be most confident in their judgments because they reached their judgments based on rational considerations and, therefore, know that they are right. On the other hand, one may argue that biased responders may be most confident in their judgments because they reached their judgments based on motivated reasoning that serves to protect their attitudes. High confidence may additionally serve the goal of protecting their attitudes. In line with the latter reasoning, we found a positive correlation between the absolute degree of bias and confidence in one’s interpretation of the pro-regulation study, *r* = .14, *p* < .001, as well as between the absolute degree of bias and confidence in one’s interpretation of the anti-regulation study, *r* = .18, *p* < .001.
